# Comparative evaluation of oral microbiologic profile in children with Type 1 Diabetes Mellitus versus healthy controls and its relation to oral health status

**DOI:** 10.1186/s12903-025-06013-2

**Published:** 2025-05-09

**Authors:** Wedad M. Nageeb, Asmaa Ali Emam Abo-Elsoud, Mona Karem Amin, Tarek Mohamed Nabil Mohamed Kamel Mahmoud, Noha El-Sayed Fathi Abdou

**Affiliations:** 1 Department of Medical Microbiology and Immunology, Faculty of Medicine, Suez Canal University, Ismailia, Egypt; 2 Department of Pediatric Dentistry, Preventive Dentistry and Dental Public Health, Faculty of Dentistry, Suez Canal University, Ismailia, Egypt; 3 Pediatrics Department, Faculty of Medicine, Suez Canal University, Ismailia, Egypt; 4 Department of Pediatric Dentistry, Preventive Dentistry and Dental Public Health, Faculty of Dentistry, Aswan University, Aswan, Egypt

**Keywords:** Diabetes mellitus, Childhood caries, Cariogenic pathogens, Oral health, CAST, Mutans streptococci, *Lactobacilli*, *Candida albicans*, *Candida dubliniensis*

## Abstract

**Background:**

Oral health is a key indicator of one’s overall health and is vitally affected by systemic diseases. A bidirectional relationship is assumed to exist between oral health and Type 1 Diabetes Mellitus. Differences in oral cariogenic microbes and their relation to metabolic control show inconsistent results. Additionally, the relation between diabetes and dental caries is inconclusive. The aim of the present study is to investigate the relation of oral health to microbiologic profile in youngsters with Type 1 Diabetes Mellitus.

**Methods:**

Sixty-three children were recruited including 22 diabetic children with poor glycemic control, 18 diabetic children with good glycemic control and 23 non-diabetic children. Oral health status was assessed using Caries Assessment Spectrum and Treatment (CAST) and oral hygiene index simplified (OHIS). Salivary and plaque samples were collected and microbiologically analyzed for identification and live colony counting *of* Mutans Streptococci, *Lactobacilli*, and different *Candida* species. The relation of different oral pathogen types and abundances with caries status and diabetes severity was assessed.

**Results:**

Salivary Mutans Streptococci were isolated at the rate of 82.5%, *lactobacilli* at the rate of 74.6%, *C. albicans* at the rate of 58.7%, and other *Candida* species collectively at the rate of 46%. The occurrence of salivary Mutans Streptococci was significantly higher in uncontrolled cases compared to healthy subjects. Salivary *C. albicans* occurred at a significantly lower frequency among controlled cases. *C. dubliniensis* and *C. tropicalis* occurred more frequently in the saliva of children with poor glycemic control. We observed higher counts of plaque Mutans Streptococci in children with poorer oral hygiene and poorer glycemic control. Both salivary and plaque *C. albicans* counts were higher in worse caries status regardless of glycemic status. Salivary *Lactobacillus* count appears as a marker of caries status.

**Conclusion:**

Although diabetes did not show significant effect on increasing risk of dental caries, the oral microbiologic profile was different among healthy and diabetic children and was affected by the level of glycemic control.

**Supplementary Information:**

The online version contains supplementary material available at 10.1186/s12903-025-06013-2.

## Background

Type 1 Diabetes Mellitus (T1DM) is a chronic metabolic disorder of elevated blood glucose levels due to lack of insulin. Worldwide prevalence of T1DM is estimated at 600,900 existing cases with annual incidence estimates at 98,200 new cases under 15 years [1]. Among children and adolescents under 19 years, 355 900 new cases are estimated with the total number expected to increase to 476 700 in 2050 [2, 3]. Diabetes is usually associated with a wide range of complications across different systems of the body and is considered a leading cause of death worldwide. Oral health is considered a key indicator of one’s overall health and general well-being. Compromised oral health is among the most important pathogenic manifestations and complications of T1DM where periodontal diseases are recognized as the sixth leading complication of Diabetes Mellitus (DM). Diabetes has been associated with different oral infections, including periodontitis, gingivitis, oral mucosal lesions, oral bacterial infections, dental caries, and candidiasis [4]. Furthermore, studies have linked poor oral hygiene to the prevalence of oral infections in diabetics. Whether oral hygiene influences microbial colonization is not fully understood [5].

A reciprocal relationship between systemic diseases and oral health is suggested, where systemic diseases like diabetes predispose to oral infections and diseases, and when oral health is compromised, oral infections can aggravate the progression of systemic diseases [6]. Inadequate dental hygiene has been proposed as a factor exacerbating diabetes progression, as the activation of proinflammatory cytokines may worsen chronic infections and contribute to the destruction of pancreatic β-cells [7]. Studying oral health and microbiologic profiles in patients with diabetes is thus of high importance.

Previous research has shown that *Streptococcus mutans* and *Lactobacillus* are the primary pathogenic bacteria in dental caries formation. In fact, *Lactobacillus* is the second most cariogenic bacterial oral flora [8]. It has been established that *Streptococcus mutans*, together with other pathogens, plays a noticeable role and acts as “core microbes” in the occurrence and development of childhood caries, where *Streptococcus mutans* most probably plays a role in the early stage of caries formation during the demineralization period [9]. Moreover, *lactobacilli* contribute to the formation of caries lesions and are crucial to the advancement of lesions, but not its initiation [10]. Polybacterial dental infections have been connected to the microbiological etiology of childhood caries. However, the current microbiological data also suggests a link between fungal pathogens and this juvenile oral disease [11]. Specifically, children with caries frequently had greater levels of *Candida* species in their oral cavities than children without caries, and the presence of fungi was positively connected with both the severity of caries and the carriage of *Streptococcus mutans* [12]. Even though fungi are commensal organisms that live within the plaque biofilm, the majority of research on caries has been on the effects of bacteria [13]. Recently, *Candida albicans* was identified as the most abundant species, followed by *Candida dubliniensis*, which was more frequently isolated in more progressive plaque communities [14].

The presence of *S. mutans* and *Lactobacillus* spp. in the oral cavity is among the important factors affecting the process of carious lesion development and the detection and enumeration of these microorganisms are very useful to identify individuals susceptible to this disease [15]. Baseline counts of *S. mutans* and *Lactobacilli* have been correlated with the risk of developing future root lesions [16]. This implies that patients who are at greater risk of developing root caries may benefit from basic microbiological screening tests. Oral levels of *Candida* have also been documented as useful indicators of microbial risk factors for caries development [17].

There is evidence that T1DM has a major role in the onset and progression of oral diseases, including periodontitis and possibly dental caries. Increased carriage of *Candida* and clinical signs of candidiasis have been linked in certain studies to decreased metabolic control, elevated blood and salivary glucose concentrations, prolonged illness duration, and the prevalence of diabetes complications. However, other studies found that T1DM did not grant a higher predisposition for yeast colonization [18]. On the other hand, numbers of bacteria belonging to the acidogenic/acid-tolerant genera *Streptococcus* and *Lactobacillus* have been found to positively correlate with increased caries incidence in people with type 2 diabetes; *Lactobacillus* bacterial numbers were particularly high among diabetics who had current dental cavities [19].

Dental caries is a multifactorial disease with many risk factors which vary in prevalence in the diabetic population. Although it may be hypothesized that diabetes increases the risk of dental caries, the relation between caries status, oral microbial alterations, oral hygiene, and T1DM remains equivocal in children and adolescents [20]. Although it is proposed that dental caries occur more frequently in diabetics, this relation is still not confirmed, and a dynamic relationship seems to exist between the oral microbiome and oral infectious diseases in T1DM [21]. The number of studies investigating oral microbial composition in T1DM patients appears to be limited when compared to T2DM patients, especially in children with contradictory findings [21]. In this study, due to paucity of data in our area, we aim to identify the prevalence of the most common etiologic pathogens incriminated in dental caries in children with type 1 diabetes mellitus and to find the relation of different pathogen types and abundances with caries status and diabetes severity. We aim to examine whether diabetic control affects oral cariogenic pathogens and caries status in children with T1DM.

## Materials and methods

### Sample size

The aim of this study was to estimate the oral microbial load in children with diabetes as compared to healthy controls. In a previous study [22], 78% of studied patients with T2DM had high counts of *Streptococcus mutans* while *M. Streptococci* compromised 8.2% in caries free teeth and 14.4% in carious children’s lesions [23]. To detect a similar effect size in our study with 90% statistical power and 5% margin of error, a minimum sample size of 28 individuals is required (*n* = 14 per group). The sample size was calculated using Open Epi, Version 3, Open-source calculator–SSCC online calculator [24].

### Ethical approval

Ethical approval for this research was obtained from the Research Ethics Committee (REC) of the Faculty of Dentistry, Suez Canal University, ethical committee approval No. 817/2024. All the steps were performed after explaining the study, all clinical examinations, and procedures to the parents and their acceptance and acquiring signed written consent from parents or legal guardians. All methods and experiments were performed in accordance with the relevant guidelines and regulations.

### Study setting and subject recruitment

A total of 63 children between the ages of 6–15 years including both boys and girls were recruited. Study subjects included 22 diabetic children with poor glycemic control (Glycosylated hemoglobin (HbA1c) ≥ 7.5%), 18 diabetic children with good glycemic control (HbA1c < 7.5%) and 23 non-diabetic children (HbA1c < 5.7%). All children with TIDM; diabetes duration of at least two years, were diagnosed and followed up at Pediatrics Diabetes and Endocrinology Clinic at Suez Canal University Hospitals. These children were followed or requested for dental care in outpatient clinic of Pediatric Dentistry Department, Faculty of Dentistry, Suez Canal University in the period between June and November 2024. Children included in the study have been diagnosed with T1DM at least 2 years prior to the examination according to the result of the glycosylated haemoglobin (HbA1c) test. Patients with any other systemic or autoimmune diseases and those with orthodontic appliances prior to examination were excluded from the study. STROBE checklist was used to ensure transparency and adherence to steps of reporting observational studies [25].

### Dental examination and oral health assessment

A comprehensive oral examination was carried out on the day of sample collection using a standard dental mirror, probe and artificial source of light on dental chair. Two independent examiners assessed the lesions and oral health status of included subjects. One of the two examiners was blinded to the diabetic status of the examined subject to avoid bias in assessment. The same examiner assessed the lesions and oral health state for more reliability. Carious lesions were detected and diagnosed using Caries Assessment Spectrum and Treatment (CAST) [26] which covers the complete range of caries lesion progression in addition to pulpal and surrounding tissue in nine codes. In order to assess the collective caries status of each subject based on CAST index, different CAST codes (levels) for each patient were converted to a collective score representing each subject by multiplying the number of teeth in each CAST code or level within each subject then summing the scores of all CAST levels observed in the same subject to have one final score per subject. The CAST code of 9 was not included in the score as it refers to others and may not represent the severe morbidity spectrum [27]. The oral health of participants was also evaluated using the Simplified Oral Hygiene Index (OHI-S) according to the criteria of Greene and Vermillion [28].

### Sample collection and microbiologic work up

#### Saliva samples

All the children were instructed not to brush their teeth the evening and the morning before sampling. Oral samples of stimulated whole saliva were stimulated by chewing paraffin gum and were secreted over a period of 5 min while being collected in a sterile container [29]. The container was sealed immediately after sample collection and refrigerated at 4 °C before transport to the laboratory at the Medical Microbiology and Immunology department. The saliva samples were then cultured after being serially diluted for bacterial species identification and enumeration.

#### Plaque and carious lesions samples

Children were instructed to refrain from oral hygiene procedures for at least 8 h prior to the collection of dental plaque samples. Using sterile periodontal curettes, material from at least two distinct tooth locations with comparable health states was combined to create each plaque sample. The samples were pooled in a sterile 1.5-ml micro-centrifuge tube with 1 ml of sterile Brain Heart Infusion Broth (BHI) (Darmstadt, Germany, Merck KGaA) [30]. The samples were then immediately transported to the laboratory no later than 4 h kept at 4 °C. Further microbiologic workup was performed at the Medical Microbiology and Immunology Department, Faculty of Medicine, Suez Canal University.

#### Identification and quantification of different Candida species

**Fig. 1:**
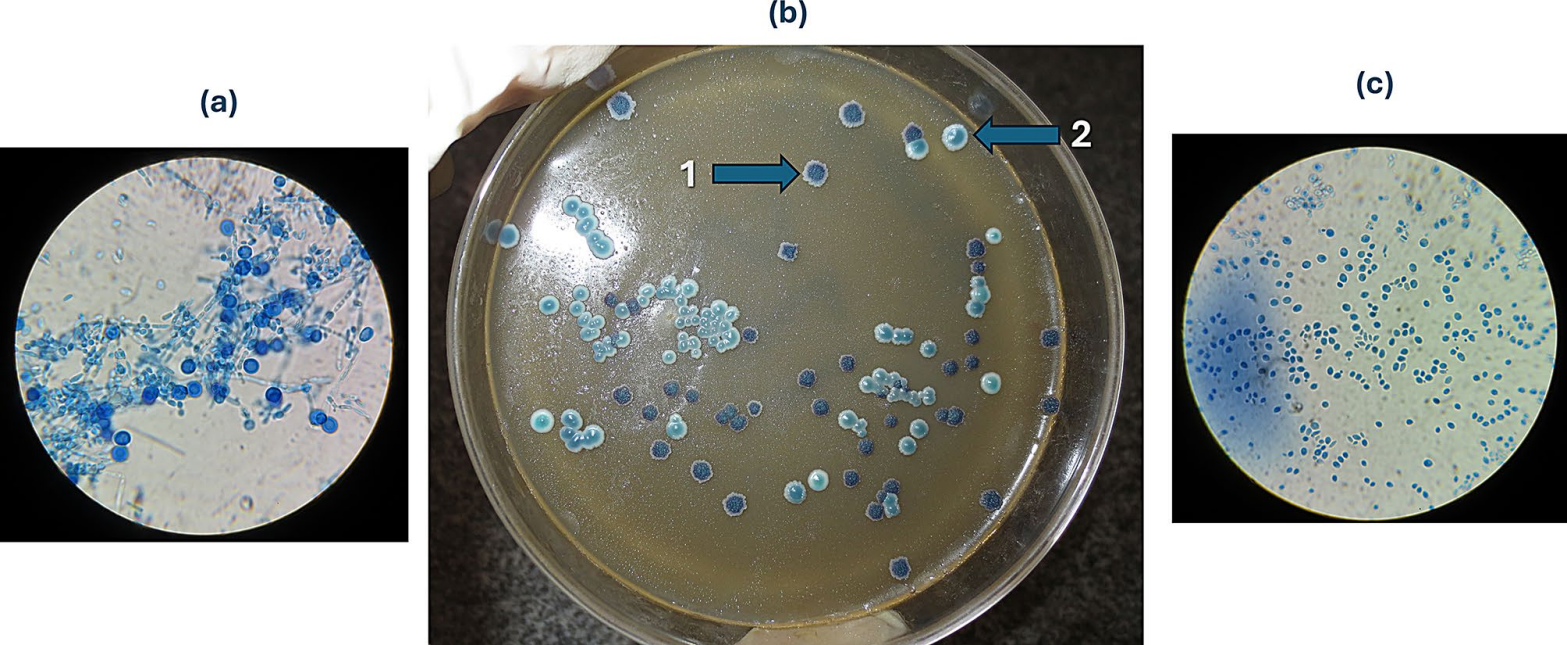
Isolation of *C. albicans* and *C. dubliniensis* on CHROMagar Candida medium supplemented with Pal’s agar. Saliva and Plaque samples were cultured for 48 h at 37 °C on CHROMagar Candida medium supplemented with Pal’s agar (**b**). The mixed media enabled discrimination of *C. albicans* smooth greenish colonies (2) and *C. dubliniensis* bluish rough colonies (1). Chlamydospore formation was detected microscopically using lactophenol cotton blue as a stain. (**a**) Colony of *C. dubliniensis stained with* lactophenol cotton blue at 100x magnification showing hyphal fringe and chlamydospore formation. (**c**) Colony of *C. albicans stained with* lactophenol cotton blue with no hyphae or chlamydospore formation

Plaque samples of 1 ml were repeatedly vortexed to allow complete elution of the dental plaque into the liquid. 0.1 ml were used for bacterial isolation by utilizing Direct streaking and spread plating techniques. Plaque samples and saliva samples were serially diluted and re-supended in 1 ml of 0.89% saline and plated onto the selective medium, Candida CHROMagar (TMMedia, India), and incubated for 72 h (37 °C) to isolate different *Candida* species. To differentiate *C. dubliniensis* from *C. albicans*, specimens were cultured on CHROMagar Candida medium supplemented with Pal’s medium which was prepared by mixing equal volumes of CHROMagar Candida medium and Pal’s agar. To prepare Pal’s agar, 50 g unsalted powdered sunflower seeds were added to 1 L of distilled water then boiled for 30 min and filtered using cheesecloth. This extract was then supplemented with 1 g of creatinine, 1 g of glucose, and 1 g of KH_2_PO_4_. After pH adjustment to 5.5, agar (15 g/liter) was added, and the mixture was autoclaved at 110 °C for 20 min. CHROMagar (TMMedia, India) Candida medium was prepared according to the manufacturer’s instructions by slowly dispersing 47.7 g of the powder medium in 1 L of purified water and bringing it to a boil by repeated heating until complete fusion of agar grains. Equal volumes of both media at their normal strength were mixed well and poured into sterile petri dishes [31]. (Fig. 1) (Supplementary Figs. 1–3). Different *Candida* species were identified by colony color and morphology on microscopic examination [32]. (Supplementary Figs. 4–7).

#### Identification and quantification of lactobacillus species

Samples were serially diluted and spread on Lactobacillus MRS agar (HiMEDIA, India). To select for growth of *Lactobacilli*, 0.2% sorbic acid was added to MRS agar. CaCo_3_ at 1.5% was also added to help obtain clear zones around colonies. Enumeration of growing colonies was performed after incubation at 37℃ at CO_2_ enriched atmosphere for 72 h (Supplementary Figs. 8 and 9) [33].

#### Identification and quantification of Mutans Streptococci

Modified Mitis Salivarius Bacitracin agar (HiMEDIA, India), containing 20% sucrose, 0.25 U bacitracin,1% tellurite, supplemented with 10 g/L colistin, 10 g/L nalidixic acid, and 4 g/L gramicidin was used as a selective medium for improved isolation of cariogenic Mutans Streptococci group. Salivary and plaque samples were serially diluted with 0.89% saline and inoculated on selective media using spiral plating. The number of Mutans Streptococci colonies on plates were counted following incubation for 72 h. Mutans Streptococci could be visually distinguished according to the colony morphology on the agar plates and also following microscopic examination (Supplementary Fig. 10) [34].

**Fig. 2:**
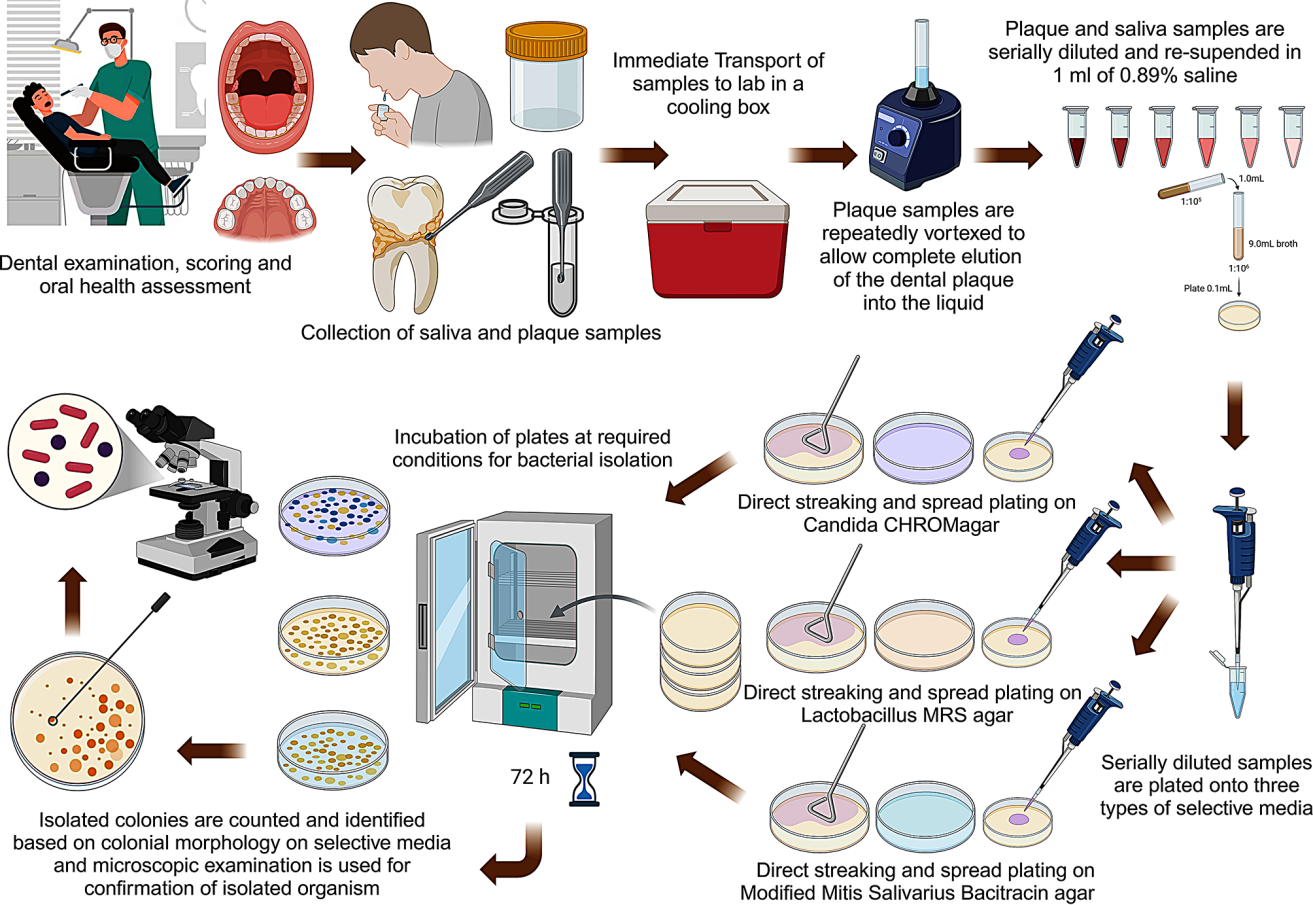
Flow chart illustrating steps of sample processing and microbiologic analysis

A flow chart summarizing sample collection, processing, and microbiologic analysis is illustrated in Fig. 2.

### Statistical analysis

A comparison of oral hygiene status and caries status among healthy children and those with good and poor glycemic control was performed using Kruskal-Wallis H tests for non-parametric data and using ANOVA test for parametric data. Chi-square test was also used to compare the difference in types of oral microbes among the three studied groups. The Kolmogorov-Smirnov test was used to assess normality of data distribution. To compare the three tested groups, Kruskal-Wallis Test was used for non-parametric data with follow up Mann-Whitney test for pairwise comparisons. A two-way Kruskal-Wallis with aligned rank transform test was then performed to find the effect of interaction of caries status and diabetes effect on counts of oral pathogens studied. To assess correlation of variables under study, Spearman rho correlation coefficient was applied. SPSS Statistical package for analysis (SPSS^®^ sofware, IBM) was used for data analysis.

## Results

### Description of study population characteristics, glycemic States, and oral health status

A total of 63 children (23 healthy children and 40 children with Type 1 DM) participated in the study. Males comprised 56.5% and 52.5% of healthy children and children with Type 1DM respectively. Of all children, 28.6% were between 6-< 9 years old, 33.3% were between 9 and 11 years old, and 38.1% were between 12 and 15 years old. The mean age of all children was 10.3 ± 2.17. The mean age was 10.5 ± 2.02 for healthy children and 10 ± 2.3 for children with Type 1 DM.

In children with T1DM, 55% had HbA1c > 7.5%, and 45% had HbA1c ≤ 7.5%. The mean HbA1c value in healthy children was 4.83 ± 0.23 (Min-Max = 4.5–5.2) while the mean HbA1c value in children with T1DM was 9.2 ± 2.5 (Min-Max = 5.7–15).

When the oral health status of included children was assessed, the mean OHIS for healthy children was 0.9 ± 0.41 (Min-Max = 0.16–2) while for children with T1DM was 0.74 ± 0.43 (Min-Max = 0–1.66) in children with good glycemic control and 0.78 ± 0.48 (Min-Max = 0.2–1.7) in children with poor glycemic control. No statistically significant difference was observed in OHIS among healthy and diabetic children with good or poor glycemic control (*p* = 0.47) (Table 1). The Median (IQR) for CAST score in healthy children was 12 (9.5), (Min-Max = 0–39) while for children with T1DM was 10.5 (17.75), (Min-Max = 0–41) in children with good glycemic control and 15.5 (5.5), (Min-Max = 0–36) in children with poor glycemic control. Kruskal-Wallis H did not show a statistically significant difference in CAST score among the three studied groups of glycemic control (*p* = 0.46) (Table 2).

**Table 1:** Group distribution of study participants based on oral hygiene index Simplified(O-H-I-S)

O-H-I-S	T1DM Uncontrolled (*n* = 22)	T1DM Controlled (*n* = 18)	Healthy (*n* = 23)	ANOVA F	Statistical Difference (*P* value)
Better (0 - < 0.7)	9	9	9	0.770	0.47
Good (0.7–1.2)	10	7	10		
Moderate (1.3-3)	3	2	4		

**Table 2:** Group distribution of study participants based on caries assessment spectrum and treatment (CAST) score

CAST Score	T1DM Uncontrolled (*n* = 22)	T1DM Controlled (*n* = 18)	Healthy (*n* = 23)	Kruskal-Wallis H	Statistical Difference (*P* value)
Better caries status (< 15)	8	11	14	1.543	0.46
Worse caries status (≥ 15)	14	7	9		

### Prevalence of cariogenic microbes studied and microbial load differences

The most prevalent microbes detected among study participants were salivary Mutans Streptococcii (52, 82.5%) and plaque Mutans Streptococci (49, 77.8%) followed by salivary *Lactobacilli* (47, 74.6%). While salivary *Candida albicans* were detected in 58.7% of cases, other salivary Candida species collectively were isolated in 46% of subjects at an overall collective rate of isolation of 61.9% with both *C. dubliniensis* and *C. parapsilosis* isolated at the rates of 19% in all study subjects. Occurrence of salivary Mutans Streptococci showed significant difference being higher in uncontrolled cases compared to healthy subjects. A statistically significant difference was also observed for the distribution of salivary *C. albicans* with observed lower occurrence of both salivary and plaque *C. albicans* in cases of controlled T1DM. The occurrence of plaque *Lactobacilli* was also observed to be lower in controlled cases when compared to both healthy and uncontrolled cases. *C. dubliniensis* occurred more frequently in saliva and plaque of uncontrolled cases. Similarly, *C. tropicalis* occurred more frequently in saliva of uncontrolled cases. The species distribution and rate of isolation of different oral microbes are shown in Table 3.

**Table 3:** Distribution of cariogenic pathogens in diabetics and non-diabetics study participants

Pathogen	T1DM Uncontrolled (*n* = 22)	T1DM Controlled (*n* = 18)	Healthy (*n* = 23)	Total (*n* = 63)	Chi-square	Statistical Difference (*P* value)
Mutans Streptococci saliva	22 (100%)	15 (83.3%)	15 (65.2%)	52 (82.5%)	14.902	0.001*
Mutans Streptococci Plaque	20 (90.9%)	13 (72.2%)	16 (69.6%)	49 (77.8%)	4.310	0.12
*Lactobacilli* saliva	18 (81.8%)	13 (72.2%)	16 (69.6%)	47 (74.6%)	1.581	0.45
*Lactobacilli* Plaque	16 (72.7%)	7 (38.9%)	13 (56.5%)	36 (57.1%)	4.745	0.09
*Candida albicans* Saliva	15 (68.2%)	6 (33.3%)	16 (69.6%)	37 (58.7%)	7.82	0.02*
*Candida albicans* Plaque	8 (36.4%)	4 (22.2%)	9 (39.1%)	21 (33.3%)	1.804	0.41
Other Candida Spp. Saliva	14 (63.6%)	7 (38.9%)	8 (34.8%)	29 (46%)	4.286	0.12
*Candida dubliniensis* Saliva	7 (31.8%)	4 (22.2%)	1 (4.3%)	12 (19%)	----------	-----------
*Candida parapsilosis* Saliva	5 (22.7%)	3 (16.7%)	4 (17.4%)	12 (19%)	----------	-----------
*Candida glabrata* Saliva	3 (13.6%)	1 (5.6%)	1 (4.3%)	5 (7.9%)	----------	-----------
*Candida tropicalis* Saliva	5 (22.7%)	1 (5.6%)	1 (4.3%)	7 (11.1%)	----------	-----------
*Candida krusei* Saliva	1 (4.5%)	0	2 (8.7%)	3 (4.8%)	----------	-----------
Other Candida Spp. plaque	7 (31.8%)	4 (22.2%)	5 (21.7%)	16 (25.4%)	0.737	0.69
Candida *dubliniensis* Plaque	6 (27.3%)	1 (5.6%)	3 (13%)	10 (15.9%)	----------	-----------
*Candida parapsilosis* Plaque	0	1 (5.6%)	1 (4.3%)	2 (3.2%)	----------	-----------
*Candida glabrata* Plaque	1 (4.5%)	1 (5.6%)	1 (4.3%)	3 (4.8%)	----------	-----------
*Candida tropicalis* Plaque	1 (4.5%)	1 (5.6%)	0	2 (3.2%)	----------	-----------
*Candida krusei* Plaque	1 (4.5%)	0	0	1 (1.6%)	----------	-----------

Kruskal-Wallis Test revealed a statistically significant difference in salivary Mutans Streptococci counts (*p* < 0.001), in plaque Mutans Streptococci counts (*p* = 0.006), in plaque *lactobacillus* counts (*p* = 0.002), and in salivary *Candida albicans* counts (*p* < 0.001) across the three studied groups (Healthy, *n* = 23: T1DM Controlled, *n* = 18: T1DM Uncontrolled, *n* = 22). Details of follow-up Mann-Whitney test pair-wise comparisons are shown in Table 4.

**Table 4:** Differences in oral microbial loads of studied cariogenic pathogens among diabetic and healthy groups

Parameter Tested	Groups Compared	Test Statistics (follow-up Mann-Whitney test)	Significance
Salivary Mutans Streptococci counts	healthy group (Md = 52800) and controlled group (Md = 3800)	U = 96.5, z = − 3.821	*p* < 0.001
Salivary Mutans Streptococci counts	uncontrolled group (Md = 46400) and controlled group (Md = 3800)	U = 137, z = − 3.654	*p* < 0.001
Plaque Mutans Streptococci counts	healthy group (Md = 10320) and uncontrolled group (Md = 29000)	U = 185, z = − 2.595	*p* = 0.009
Plaque Mutans Streptococci counts	controlled group (Md = 9400) and uncontrolled group (Md = 29000)	U = 183, z = − 2.809	*p* = 0.005
Plaque Mutans Streptococci counts	group with better oral hygiene (Md = 4500) and the lower hygiene group (Md = 33500)	U = 451, z = − 3.856	*p* < 0.001
Plaque *lactobacillus* counts	healthy group (Md = 1250) and uncontrolled group (Md = 4250)	U = 207.5, z = − 2.269	*p* = 0.02
Plaque *lactobacillus* counts	controlled group (Md = 600) and uncontrolled group (Md = 4250)	U = 97, z = − 3.259	*p* = 0.001
Salivary *Lactobacillus* counts	healthy group (Md = 12160) and uncontrolled group (Md = 5500)	U = 218, z = − 2.075	*p* = 0.04
Salivary *Lactobacillus* counts	group with better caries status (Md = 6400) and the worse caries status group (Md = 9900)	U = 482.500, z= -2.015	*p* = 0.04
Salivary *Candida albicans* counts	healthy group (Md = 2000) and controlled group (Md = 11200)	U = 127.5, z = − 3.853	*p* < 0.001
Salivary *Candida albicans* counts	healthy group (Md = 2000) and uncontrolled group (Md = 400)	U = 157.5, z = − 3.308	*p* = 0.001
Salivary *Candida albicans* counts	controlled group (Md = 11200) and uncontrolled group (Md = 400)	U = 27.5, z = − 5.687	*p* < 0.001
Plaque *Candida albicans* counts	group with better oral hygiene (Md = 11350) and the lower hygiene group (Md = 18400)	U = 520, z = − 2.437	*p* = 0.02

### The relationship of microbial counts to oral health status and glycemic status

To investigate the relation of caries status to the counts of different species studied, patients were divided into a group with better caries status with CAST score < 15 and another group with Worse caries status with CAST score ≥ 15. A statistically significant difference was only found in salivary *Lactobacilli* counts.

**Fig. 3:**
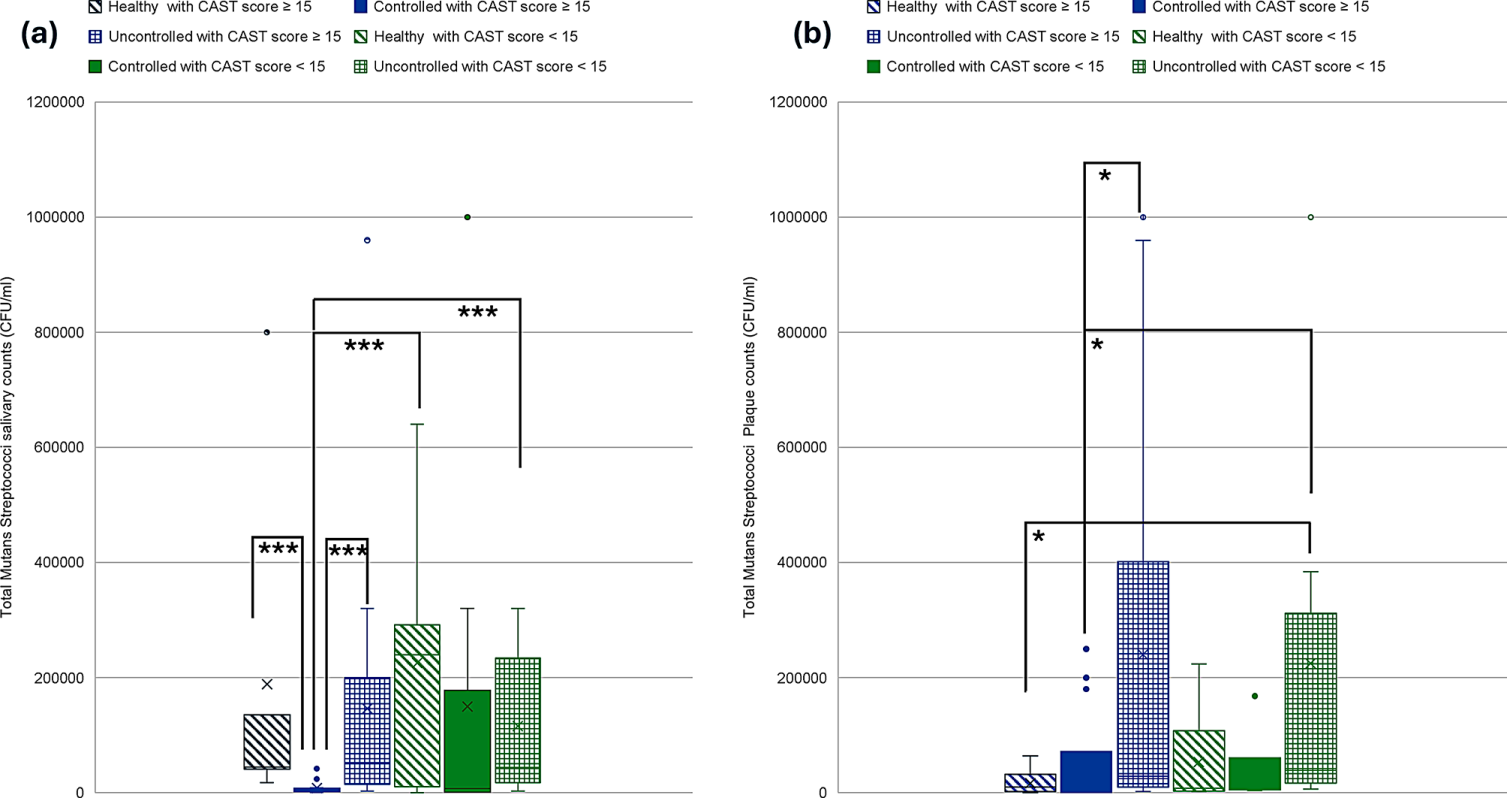
Distribution of Mutans Streptococci counts in relation to different glycemic states and caries status. **a**. Mutans Streptococci salivary counts. **b**. Mutans Streptococci plaque counts. Blue color refers to CAST score ≥ 15, Green color refers to CAST score < 15, ***≤0.001, **p ≤ 0.01, *p < 0.05

**Fig. 4:**
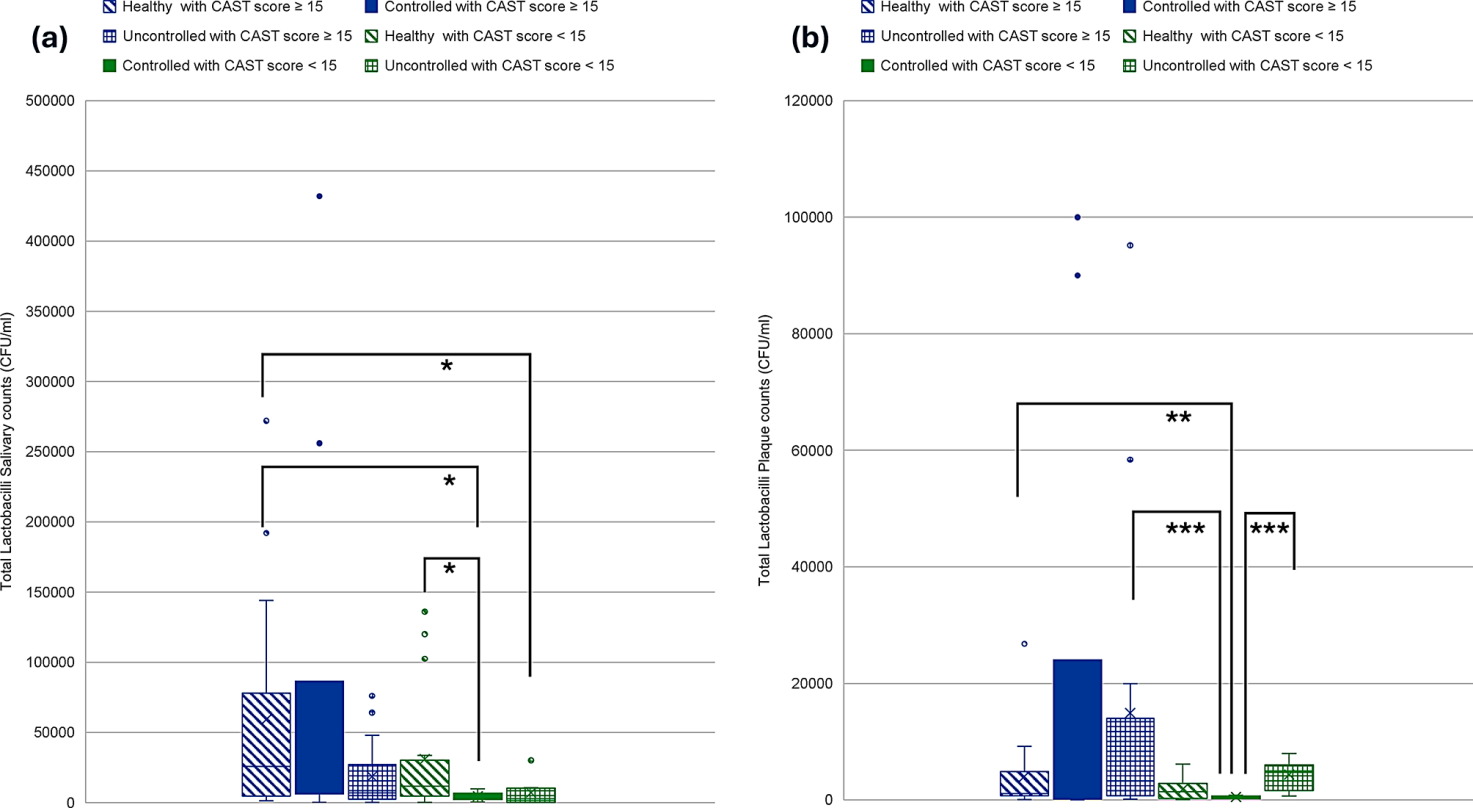
Distribution of *Lactobacilli* counts in relation to different glycemic states and caries status. **a**. *Lactobacilli* salivary counts. **b**. *Lactobacilli* plaque counts. Blue color refers to CAST score ≥ 15, Green color refers to CAST score < 15, ***≤0.001, **p ≤ 0.01, *p < 0.05

**Fig. 5:**
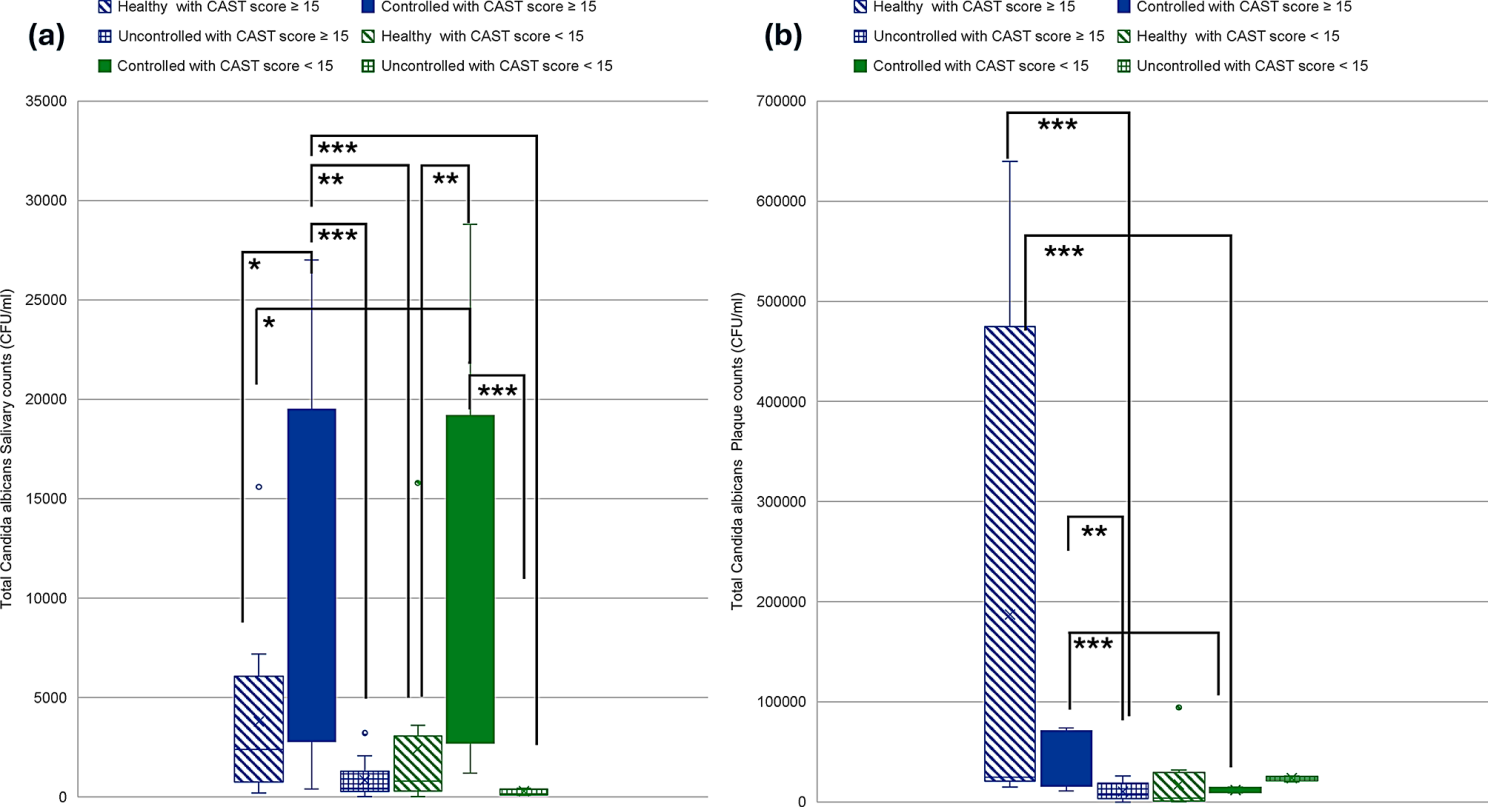
Distribution of *Candida albicans* counts in relation to different glycemic states and caries status. **a**. *Candida albicans* salivary counts. **b**. *Candida albicans* plaque counts. Blue color refers to CAST score ≥ 15, Green color refers to CAST score < 15, ***≤0.001, **p ≤ 0.01, *p < 0.05

A two-way Kruskal-Wallis with aligned rank transform test was performed to test the interaction of caries status and diabetes effect on counts of oral pathogens studied. Kruskal-Wallis H showed significant differences in ranks of salivary Mutans Streptococci counts (*p* = 0.001), plaque Mutans Streptococci counts (*p* = 0.02), salivary *Lactobacilli* counts (*p* = 0.04), plaque *Lactobacillus* counts (*p* = 0.007), salivary *Candida albicans* counts (*p* < 0.001) and plaque *Candida albicans* counts (*p* < 0.001) (Figs. 3, 4 and 5). Salivary Mutans Streptococci counts do not appear to reflect the caries status and appear to be more affected by glycemic control status regardless of caries status (Fig. 3). Additionally, Mutans Streptococci plaque counts appear to be more affected by state of glycemia especially with worse caries status (Fig. 3). Salivary *lactobacilli* may be affected by glycemic state being higher in euglycemic subjects and is also higher in cases with worse caries condition while plaque lactobacilli count appear to be less affected by glycemic state or caries state (Fig. 4). Salivary *C. albicans* counts were generally lower than plaque counts and did not appear to be related to glycemic state. Plaque candida albicans counts appear higher in worse caries status regardless of glycemic status (Fig. 5).

**Fig. 6:**
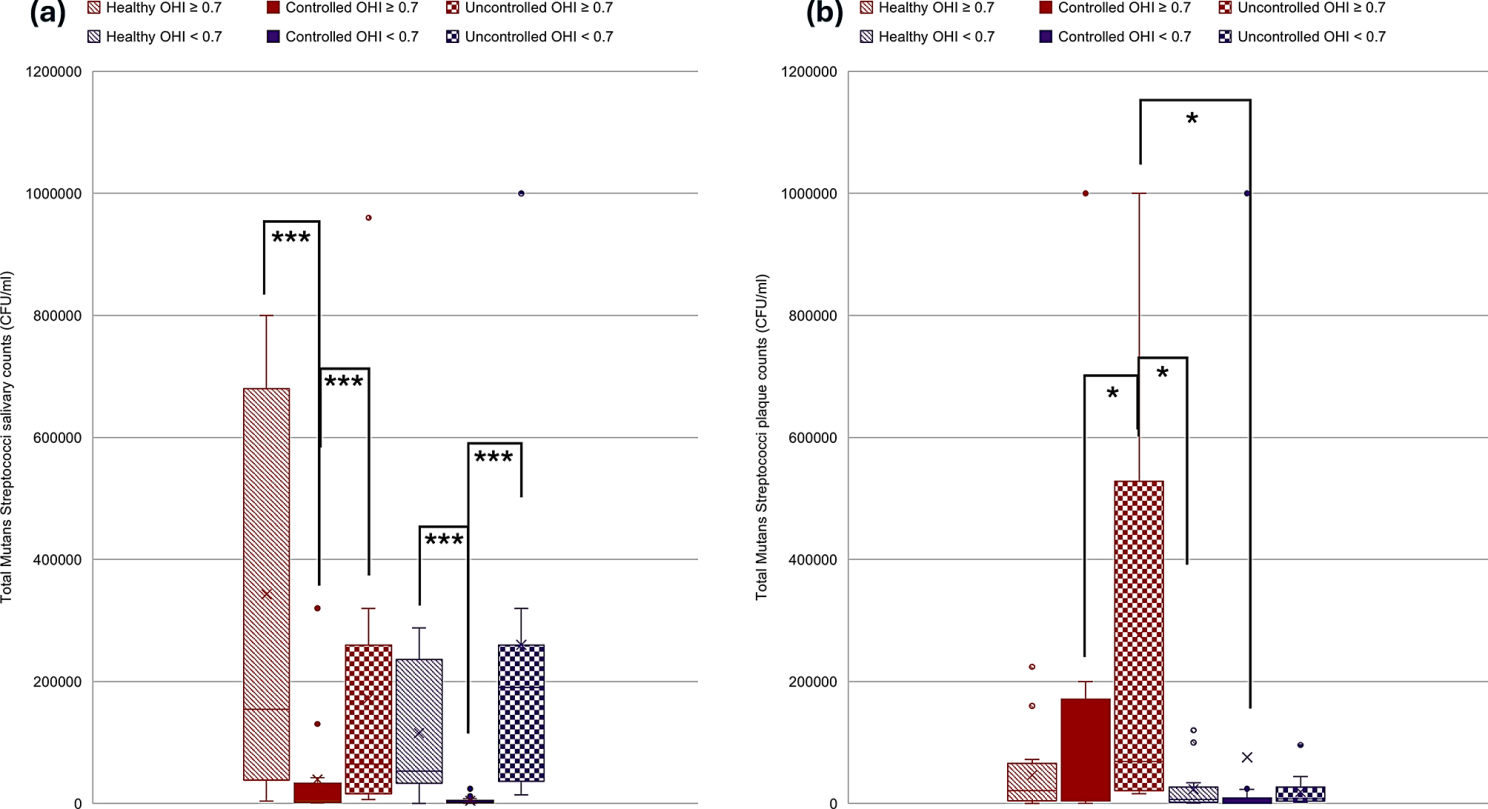
Distribution of Mutans Streptococci counts in relation to different glycemic states and oral hygiene status. **a**. Mutans Streptococci salivary counts. **b**. Mutans Streptococci plaque counts. Red color refers to OHI score ≥ 0.7, Violet color refers to OHI score < 0.7, ***≤0.001, **p ≤ 0.01, *p < 0.05

**Fig. 7:**
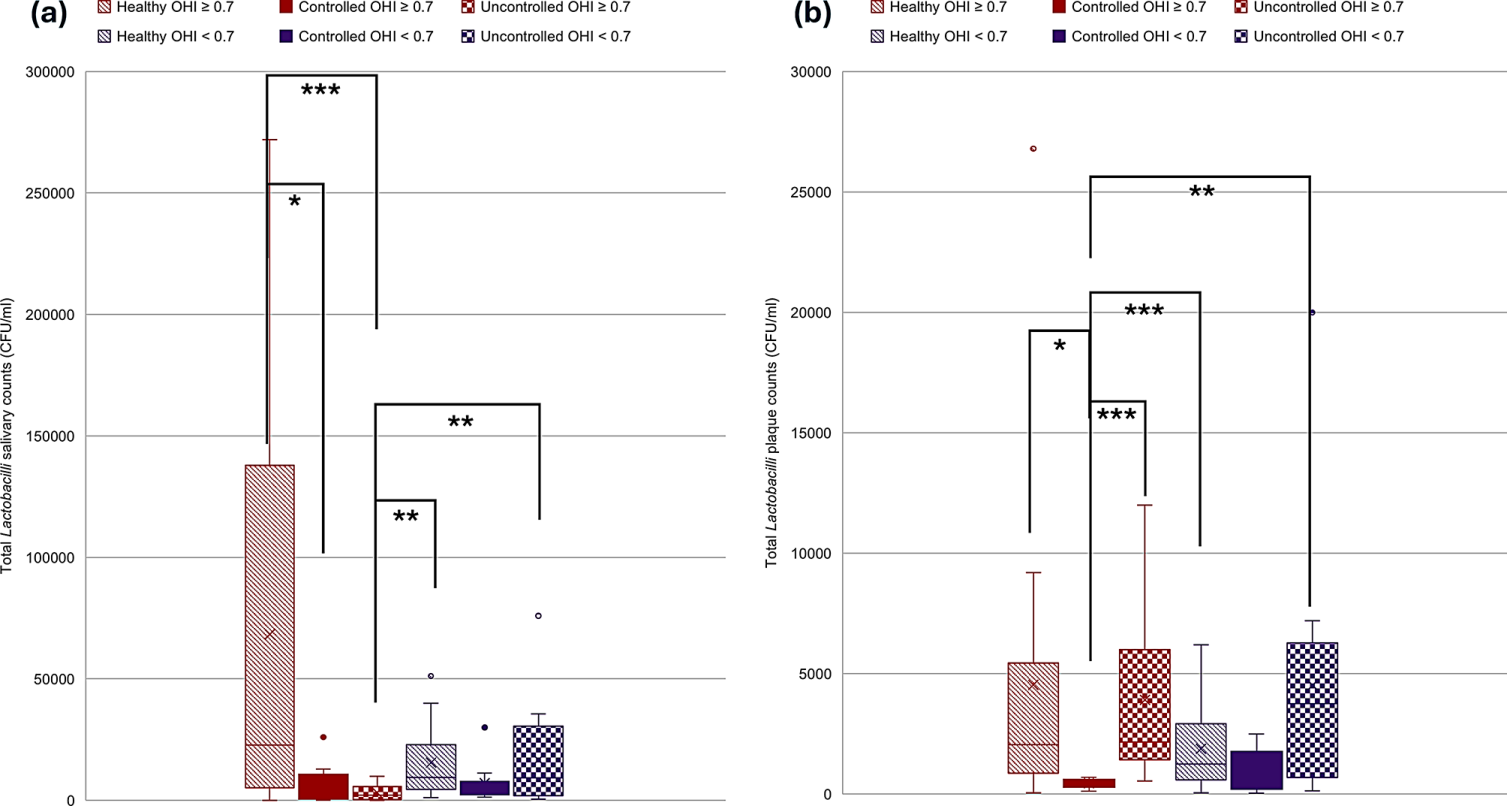
Distribution of *Lactobacilli* counts in relation to different glycemic states and oral hygiene status. **a**. *Lactobacilli* salivary counts. **b**. *Lactobacilli* plaque counts. Red color refers to OHI score ≥ 0.7, Violet color refers to OHI score < 0.7, ***≤0.001, **p ≤ 0.01, *p < 0.05

**Fig. 8:**
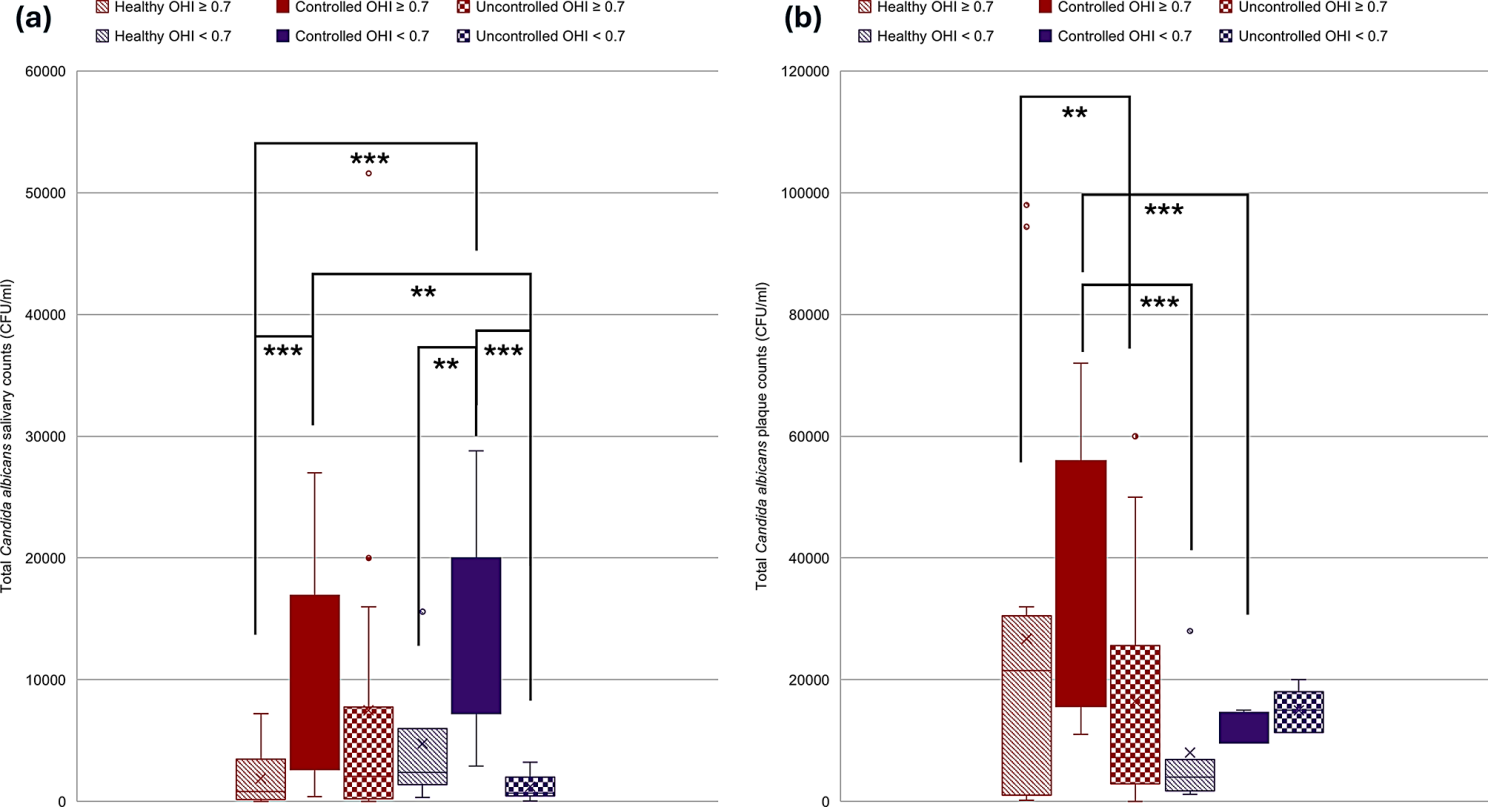
Distribution of *Candida albicans* counts in relation to different glycemic states and oral hygiene status. **a**. *Candida albicans* salivary counts. **b**. *Candida albicans* plaque counts. Red color refers to OHI score ≥ 0.7, Violet color refers to OHI score < 0.7, ***≤0.001, **p ≤ 0.01, *p < 0.05

To investigate the relation of oral hygiene to the counts of different species studied, patients were divided into a group with better oral hygiene status with OHIS < 0.7 and another group with good to moderate status with OHIS ≥ 0.7. A statistically significant difference was found in both plaque Mutans Streptococci counts and plaque *C. albicans* counts. A two-way Kruskal-Wallis with aligned rank transform test was performed to test the effect of interaction of oral hygiene and diabetes effect on counts of studied oral pathogens. Kruskal-Wallis H showed significant differences in ranks of salivary and plaque Mutans Streptococci counts, salivary and plaque *Lactobacillus* counts, and also in salivary and plaque *C. albicans* counts with *p* values of < 0.001, 0.01, 0.002, 0.001, < 0.001, and 0.008 respectively (Figs. 6, 7 and 8). Figure 6 shows that the controlled group has demonstrated significantly the lowest salivary Mutans Streptococci counts compared to both healthy and uncontrolled groups. Figure 8 shows that the salivary Candida counts are higher in the controlled group than salivary counts in both healthy and uncontrolled groups regardless of the oral hygiene level. Figure 8 also shows that plaque Candida levels may be affected by both the degree of glycemic control and also oral hygiene status.

### Correlation between oral microbial counts and oral hygiene and glycemic state

The overall relationships between oral microbial loads, scores of oral hygiene and HBA1c levels were investigated using Spearman rho correlation coefficient. There was a significant medium positive correlation between plaque Mutans Streptococci counts and HBA1c levels, *r* = 0.310, *p* = 0.01. *Lactobacillus* plaque counts and *C. albicans* salivary counts also showed a moderate positive correlation with CAST score, *r* = 0.33 (*p* = 0.008) and *r* = 0.379 (*p* = 0.002) respectively.

## Discussion

### Relationship between diabetes and oral health status

There is evidence that T1DM plays a role in the onset and progression of different types of oral diseases, including dental caries and periodontal diseases. Patients with diabetes may experience diminished resistance to infection because of tissue metabolic imbalances or changes in the oral microflora. In this study, we aim to explore the possible load and distribution of the most common oral pathogens and their relation to glycemic control, caries status, and oral health status in children with T1DM. Although some studies find higher predisposition to caries and periodontal diseases in diabetic patients [35–37], other studies did not demonstrate any difference in periodontal or microbiologic status in young adults with Insulin Dependent Diabetes Mellitus IDDM compared to healthy controls [38]. Like our study, other studies have not identified significant differences in oral health status between diabetic children and their healthy counterparts [39] and even more surprisingly, some other studies have detected oral health values of children with DM as better than national averages [40].

In the current study, no statistically significant difference was observed in OHIS or in Caries status as assessed using CAST score among healthy and diabetic children with good or poor glycemic control. This finding may be supported by other studies showing similar prevalence of dental caries among healthy and diabetic children [35, 41, 42]. This finding is contradictory to findings from other studies showing that children with T1DM are more prone to developing oral diseases as compared to healthy children [43]. Conflicting evidence exists in the literature regarding the different prevalences of caries among healthy and diabetic children with variable methodologies and variable levels of evidence available [44]. The current study found no significant differences in caries status between diabetic subjects and healthy controls, as evidenced by other studies [45, 46].

### Oral microbiologic profile in children with T1DM

The oral microbiologic profile showed differences in species distribution and counts among diabetic children and healthy controls. Mutans Streptococcus, *Lactobacilli* and *Candida spp* have been identified in other studies as the predominant microbes from saliva of diabetes patients [47].

In our study, Mutans Streptococci were isolated at the rate of 82.5% among all study subjects which is slightly lower than rates identified in other studies showing *Streptococcus spp* isolation at the rate of 99.6% [5]. Other studies showed a lower overall rate of Streptococcal isolation at 66.6% with a significant difference in their counts among healthy and diabetic groups which was similar to our results [18]. Another study has shown a rate of 49.4% *Streptococcus mutans* isolation with significantly higher occurrence among children with poor glycemic control compared to those with good glycemic control which is similar to our findings [48].

Other studies have isolated *C. albicans* at the rate of 17.0% while other *Candida spp* were isolated at the rate of 6.8% [5]. In the same study, non-albicans *Candida spp* were more prevalent in diabetic patients than non-diabetics [5]. Our study showed similar results in which non-albicans *Candida spp* were more prevalent in diabetic subjects compared to non-diabetics and especially more prevalent in uncontrolled diabetic cases. Salivary and plaque *C. albicans* were isolated at the overall rates of 58.7% and 33.3% respectively while other non-albicans *Candida spp.* were isolated from saliva and plaque at the rates of 46% and 25.4% respectively. In this study, Salivary *C. albicans* was isolated at the rate of 68.2% in children with uncontrolled diabetes and at the rate of 33.3% of those with controlled diabetes. This is similar to results from other studies showing an elevated frequency of *C. albicans* detection among children with poor glycemic control [18]. Plaque *C. albicans* were recovered at the rate of 36.4% in uncontrolled patients and at the rate of 22.2% from controlled subjects. These rates are lower compared to other previous studies reporting isolation rates of 85% recovered from insulin-treated diabetic patients [49]. In other studies, oral *C. albicans* was isolated at the rates of 18.6%, 30%, and 33.3% of healthy non-diabetic control subjects respectively [13, 18, 50]. The rate of oral carriage of *Candida* across different studies varied widely between 18–80% [51] and ranged from 7.7 to 78% according to a recent meta-nalysis study [52]. In other studies [13, 50, 53], a significant difference was identified in the isolation of *Candida* between diabetics and healthy controls attributed to the effect of hyperglycemia. However, in our current study significant difference in rate of *C. albicans* isolation was observed between controlled diabetic cases and both healthy and uncontrolled being significantly lower in controlled cases.

Similar to another previous study [49], which first reported the isolation of *C. dubliniensis* from diabetic patients, *C. dubliniensis* was the second most common isolated species next to *C. albicans* in diabetic patients treated with insulin (50%). This was followed by *C. glabrata* (20%), then *C. tropicalis* and *C. parapsilosis* at similar rates of 10%. In the current study, salivary *C. dubliniensis* was isolated at the rate of 27.5% of diabetic patients with higher rate of isolation among uncontrolled diabetics (31.8%). This was followed by salivary *C. parapsilosis* isolated at the rate of 20% among diabetic patients. Salivary *C. tropicalis* was isolated at the rate of 15% of diabetic subjects and the salivary *C. glabrata* at the rate of 10% of diabetics. Other studies have similarly reported multiple isolation of non-albicans species from diabetic patients including *C. glabrata* and *C. tropicalis* [54]. *C. dubliniensis* has been isolated from the oral cavities of 58 out of 318 (18.2%) insulin-treated diabetic patients in another study [55]. Other studies have also reported the predominance of *C. dubliniensis* as non-albicans species, followed by *C. glabrata*, *C. kruseii* and *C. parapsilosis* in diabetic patients [51]. This is similar to the findings from our current study.

### *Candida albicans* relation to oral health and glycemic States

Although the underlying mechanism is not well-established, some studies have proposed higher susceptibility to oral candidiasis in diabetic patients due to the higher glucose levels in saliva that may favor fungal proliferation. It is proposed that high salivary glucose levels can result in the glycosylation of proteins during hyperglycemia peaks and the adherence of fermentation products, which may be a necessary initial step in *Candida* colonization and subsequent infection. There may eventually be more receptors available for *Candida* as a result of these products. Thus, the initial mechanism of colonization is more likely to occur in diabetics and to occur with greater intensity [56]. On the contrary, other studies have failed to demonstrate a relationship between salivary glucose concentration and oral candidal carriage [56].It is important to consider that other host factors can also affect candidal carriage rate including candidacidal activity of salivary neutrophils, secretory immunoglobulin A, and salivary flow rate which all are commonly altered in diabetic patients. In addition, the effect of other factors including the state of metabolic controls and dietary habits should also be considered.

In this study, Salivary counts of *C. albicans* may not reflect degree of glycemic control, however, it showed positive moderate correlation with caries status as assessed using CAST score. On the other hand, plaque *C. albicans* levels appear to be more affected by oral hygiene status and caries status than being correlated to the level of glycemia indicating a possible significant role in caries. Similar to our results, another previous study has also identified lack of relation between oral candidal load and glycemic control [49]. Other studies have also shown a lack of significant differences in oral Candida counts among healthy children and those with different levels of glycemic control [57]. Regarding caries risk, a recent meta-analysis reports that when compared to people without these oral cavity microbes, children and adolescents with *Candida* spp. had a higher rate of dental caries with a prevalence rate ranging between 27.2 and 100%. Prevalence of dental caries was 80% higher in children harboring oral *Candida spp.* [52]. This supports findings from our study that show significantly higher counts of plaque *C. albicans* in children with lower levels of oral hygiene and in children with worse caries status and higher CAST scores.

### Mutans Streptococci relation to oral health and glycemic States

Salivary Mutans Streptococci levels were highest in the healthy group compared to diabetic group which did not appear to be related to oral hygiene levels. Controlled glycaemia showed the lowest levels of salivary Mutans Streptococci regardless of the level of oral hygiene and regardless of caries status. This agrees with results from other studies showing lower levels of Mutans Streptococci in well-controlled diabetes [58, 59]. This is also supported by findings from another study showing significant difference in Mutans Streptococci counts among healthy and diabetic children and also among well-controlled and poorly controlled glycaemia [46].

Plaque Mutans Streptococci levels showed significant relation to both oral hygiene status and to glycemic control and was significantly higher with poor glycemic control and in worse oral hygiene levels. Plaque Mutans Streptococci counts showed a moderate positive correlation with HBA1c level. This may be supported by findings from other studies that showed that the prevalence of *S. mutans* was related to poor glycemic control [48, 60]. On the contrary, other studies failed to find statistically significant difference in salivary Mutans Streptococci levels among healthy and diabetic children in addition to children with different levels of glycemic control [57].

These findings indicate that levels of plaque Mutans Streptococci may reflect both glycemic status and caries status being higher in higher CAST scores and lower oral hygiene with poor glycemic control. This is supported by the known observation that Mutans Streptococci continues to be the most common cariogenic species found with its well-known role in initiating caries process in acidogenic plaque [61]. Studies have demonstrated significant detection of plaque Mutans Streptococci in young children with severe childhood caries compared to caries-free children [62].

The findings of higher Mutans Streptococci count with poor glycemic controls may be supported by the fact that high glucose in oral fluids helps bacterial proliferation and increases dental plaque formation, probably leading to the higher incidence of caries in poorly controlled diabetes as compared to controlled diabetes [42]. As a result of higher glucose levels, a reduction in salivary PH is also expected, leading to an expected higher activity of cariogenic bacteria, including Streptococci, among others [45]. The proliferation of these species will result in an even lower pH, perpetuating a vicious cycle that also affects the growth of protective microflora in the oral cavity [56, 63]. However, differences in findings from different studies may be attributed to other factors in addition to the state of glycaemia, including dietary habits and oral hygiene practices, which are not usually assessed in all studies. With adequate metabolic control, salivary changes including high glucose levels and low PH are prevented leading to deceleration of acidogenic bacterial flora proliferation and decreased plaque formation [42].

### *Lactobacilli* relation to oral health and glycemic States

In the current study, Salivary *lactobacillus* count revealed the highest occurrence in euglycemic saliva compared to diabetics. This is supported by findings from other studies showing that oral *Lactobacilli* counts were significantly higher in healthy children compared to diabetic children [64] However, this is contrary to results from other studies showing tendency towards higher *Lactobacilli* counts in poorly controlled diabetes [58, 59]. Findings from other studies failed to show significant difference in salivary l*actobacilli* levels among healthy and diabetic children with different glycemic states [57]. Other studies have shown a lack of significant difference in salivary *Lactobacilli* counts among non-diabetic and diabetic group, however, a difference was observed between good and bad levels of glycemia [46].

When CAST score was used to assess caries status and not considering glycemic state, only salivary *lactobacilli* among the studied oral microbes showed significant relation to caries status. This may reflect the important role of salivary *lactobacilli* in caries progression. Additionally, plaque lactobacilli showed moderate positive correlation to caries status as assessed using CAST score.

Although salivary counts were higher in euglycemic saliva, plaque *lactobacilli* were significantly higher in diabetic group. From the current study results, it appears that higher glycemic state increase plaque lactobacillus numbers probably indicating a different metabolomic profile affecting its adherence and establishment on tooth surface. Higher glycemic states appear to facilitate sustained colonization of lactobacilli and somehow offer a retentive niche. This may be specifically enhanced by the greater capacity of *Lactobacilli* to tolerate acidic environments and low PH compared to *S. mutans* [65] The exact mechanism requires further investigation.

### Strengths and limitations

Oral microbial composition and oral infectious diseases in patients with T1DM seem to be governed by different factors being mainly affected by shifts in oral immune responses and also by shifts in metabolic controls. The collective oral microbiota dysbiosis rather than a single pathogen appears to play a greater role in contributing to various oral and systemic diseases [66, 67].The relationship between diabetes and oral health state is assumed to be a two-way relationship with reciprocal feedback with oral microbiota and immunological dysregulation acting as key role players [66, 68].

The current study has assessed the three most common cariogenic pathogen species, shedding light on different *Candida* species that are usually less commonly assessed or regarded. It uses the conventional microbial culture-based colony counting method, which, despite being labor-intensive is considered the golden standard and more sensitive than other more advanced molecular methods. The study has also evaluated microbial communities in both plaque and saliva, a feature often overlooked in previous research. All these points are considered strengths of the current study.

Food habits, salivary characteristics, and antibiotic treatment regimen all affect the diversity and numbers of microflora. Poor oral health may unfavorably affect glycemic control and the incidence of medical complications in diabetic patients [69]. Some studies have proposed that dietary habits including lower consumption of carbohydrates due to a greater concern with health status in diabetic subjects may underly the lack of correlation of glycemic status and caries status. This suggests that proper dietary habits and metabolic control can potentially reduce the incidence of caries in children with T1DM.

In addition, it is also important to consider the multifactorial nature of caries etiology that includes many diverse factors in addition to the diverse cariogenic microbiome. Numerous factors, including host genetics, salivary composition, tooth sensitivity to acid demineralization, nutrition, fluoride exposure, tooth anatomy, and enamel composition, all interact with the plaque microbiome to determine caries risk [61], which may also interpret the lack of significant difference in caries status due to diabetes alone. A possible limitation of the current study is the presence of confounding factors that may affect the relation of diabetes to oral microbial loads and health status. These confounding factors including dietary habits, oral hygiene practices, nutrition, and fluoride exposure have not been assessed in the current study. The fact of having similar levels of oral hygiene to healthy children in the current study and other studies with similar findings may be attributed to sucrose restricted diets consumed by Diabetic children and probably a better adherence to oral brushing habits which highlights that control of oral pathology in diabetics is achievable. The current study findings may underscore the importance of different other factors in contributing to the risk of caries in children [70]. To the best of our knowledge, HbA1c is the best used classifier for glycemic control, however, possible bias may be encountered in its consistent ability to reflect metabolic control. Another limitation of the study is the assessment of the three most common oral cariogenic pathogens. It is crucial to acknowledge that the variety of species constituting the oral microbiome and their functional interactions with the predominant oral pathogen may influence the relationship between diabetes and caries status, potentially playing a significant role in modifying this interaction. Based on that, a broader diversity of microbes may be included to investigate such interaction in future similar studies.

## Conclusion

From the study findings, diabetes does not show a significant effect on increasing risk of dental caries, however, the oral microbiologic profile was different among healthy and diabetic children. In those children with worse oral hygiene and poorer glycemic control, higher counts of plaques Mutans Streptococci were observed. Salivary Mutans Streptococci counts did not reflect the caries status and were more affected by glycemic control status. Both salivary and plaque *C. albicans* counts were higher in worse caries status regardless of glycemic status. Salivary *lactobacillus* levels were higher in euglycemic saliva. Salivary *lactobacilli* levels appear to be the best marker reflecting caries status regardless of glycemic status. Plaque *Lactobacilli counts* demonstrates positive correlation with caries status.

Having this evidence, adequate metabolic control, routine oral health examination and oral microbial load monitoring are recommended as a multidisciplinary approach in diabetic patients to prevent and retard diabetes-related health complications.

## Electronic supplementary material

Below is the link to the electronic supplementary material.

Supplementary Material 1

## Data Availability

All data generated and analyzed in this work are presented in manuscript and its associated supplementary information.

## Abbreviations

T1DM: Type 1 Diabetes Mellitus
CAST: Caries Assessment Spectrum and Treatment
OHIS: Oral Hygiene Index Simplified
BHI: Brain Heart Infusion
IDDM: Insulin Dependent Diabetes Mellitus
DM: Diabetes Mellitus
HbA1c: Glycosylated hemoglobin
