# Supplementary material for: Comparative evaluation of oral microbiologic profile in children with Type 1 Diabetes Mellitus versus healthy controls and its relation to oral health status
Source: BMC Oral Health. 2025 May 9;25:698. doi: 10.1186/s12903-025-06013-2 (PMC12065165; doi:10.1186/s12903-025-06013-2)
Supplement: Supplementary file 1 — Supplementary Material 1 [file 12903_2025_6013_MOESM1_ESM.pdf]

Comparative Evaluation of Oral Microbiologic Profile in Children with Type 1 Diabetes Mellitus Versus Healthy Controls and Its Relation to Oral Health Status

Wedad M. Nageeb[1*], Asmaa Ali Emam Abo-Elsoud [2], Mona Karem Amin [3], Tarek Mohamed Nabil Mohamed Kamel Mahmoud [2], Noha El-Sayed Fathi Abdou [4]

[1]Department of Medical Microbiology and Immunology, Faculty of Medicine, Suez Canal University, Ismailia, Egypt

[2]Department of Pediatric Dentistry, Preventive Dentistry and Dental Public Health, Faculty of Dentistry, Suez Canal University, Ismailia, Egypt

[3]Pediatrics Department, Faculty of Medicine, Suez Canal University, Ismailia, Egypt,

[4]Department of Pediatric Dentistry, Preventive Dentistry and Dental Public Health, Faculty of Dentistry, Aswan University, Aswan, Egypt

***Correspondence: Wedad M. Nageeb,** Department of Medical Microbiology and Immunology, Faculty of Medicine, Suez Canal University, Ismailia, Egypt

E-mail: wedad_saleh@med.suez.edu.eg

Supplementary Figure 1: Microscopic picture of *Candida albicans*

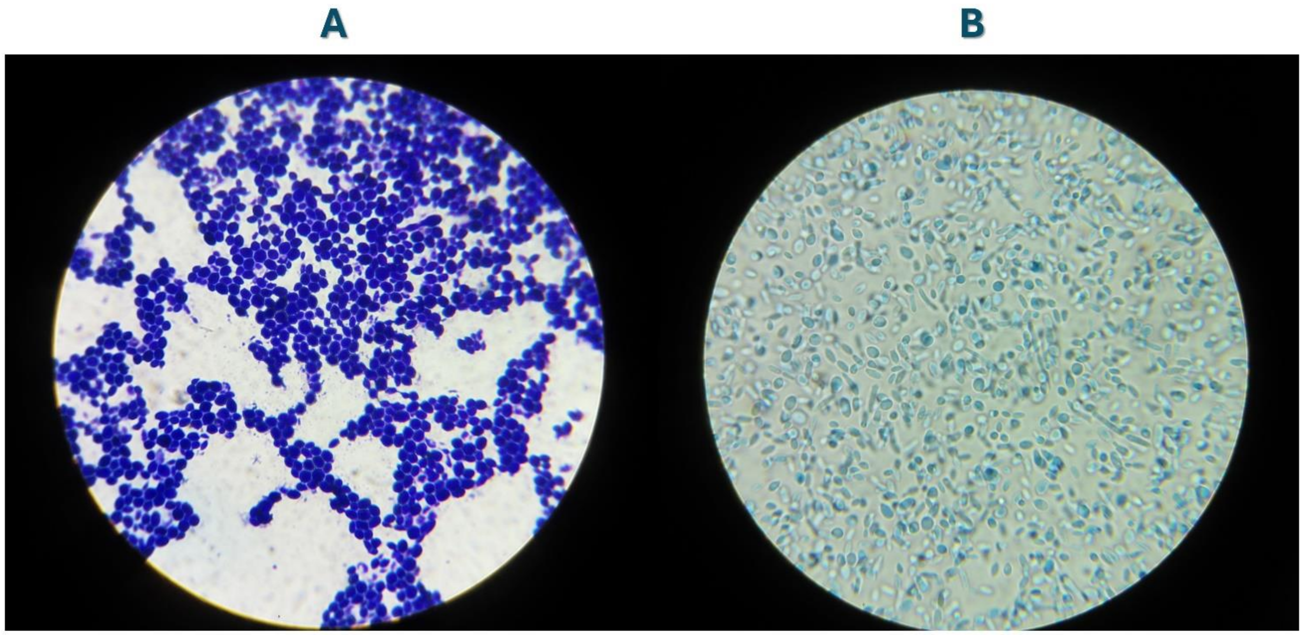

(A) *Candida albicans* morphology at 100x magnification using crystal violet stain showing round to oval large cells retaining the CV stain. (B) *Candida albicans* morphology at 100x magnification using lactophenol cotton blue showing pleomorphic cells with larger blastoconidia 4-6 um with pseudohyphae.

Supplementary Figure 2: Microscopic picture of *Candida dubliniensis*

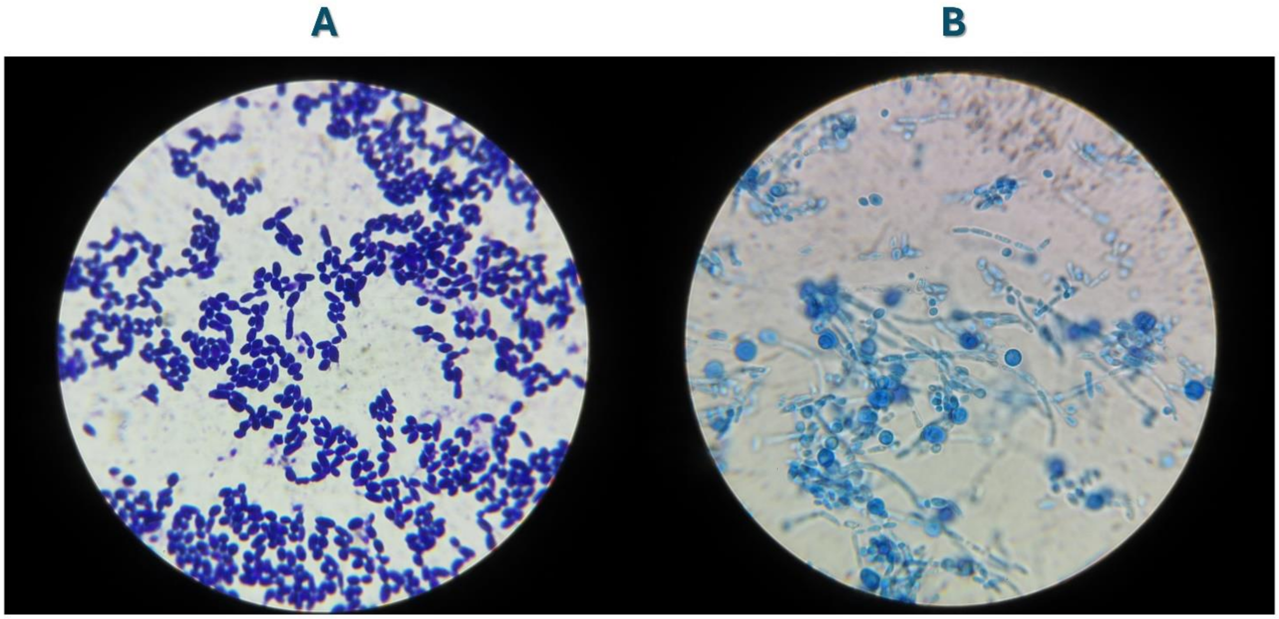

(A) *Candida dubliniensis* morphology at 100x magnification using crystal violet stain showing oval sightly elongated cells with budding. (B) *Candida dubliniensis* morphology at 100x magnification using lactophenol cotton blue showing oval cells with chlamydospore and hyphae.

Supplementary Figure 3: Serial dilution cultures of *Candida albicans* on CHROMagar Candida medium

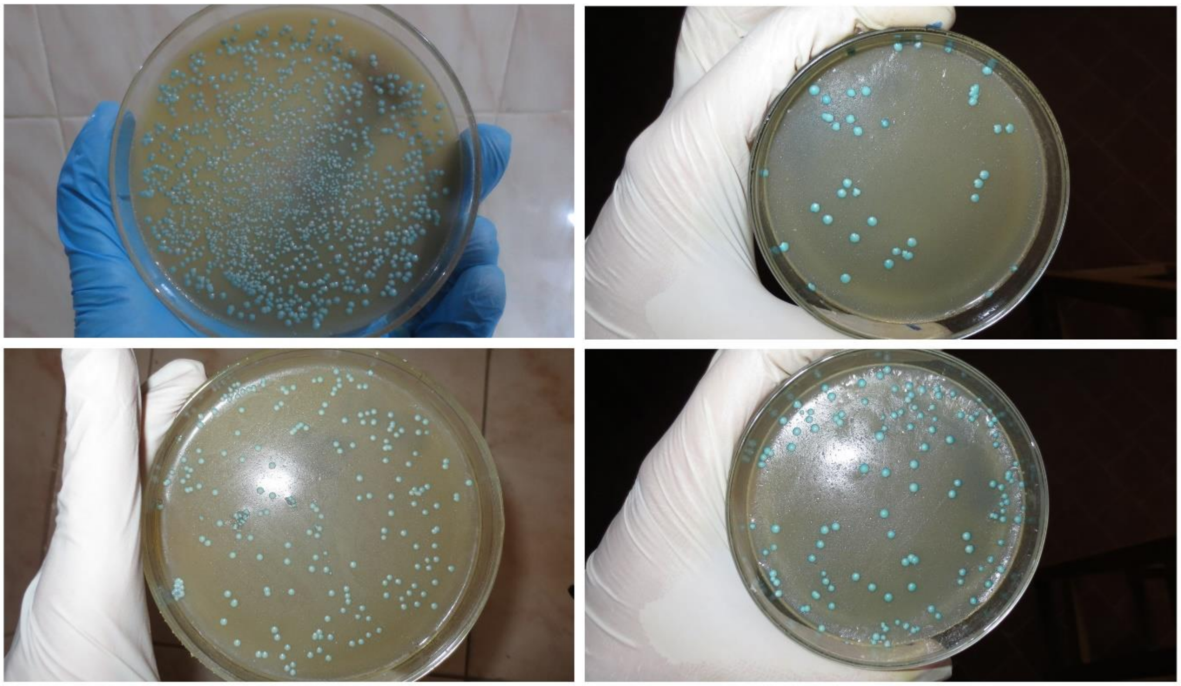

Supplementary Figure 4: Macroscopic and Microscopic morphology of isolated *C. glabrata*

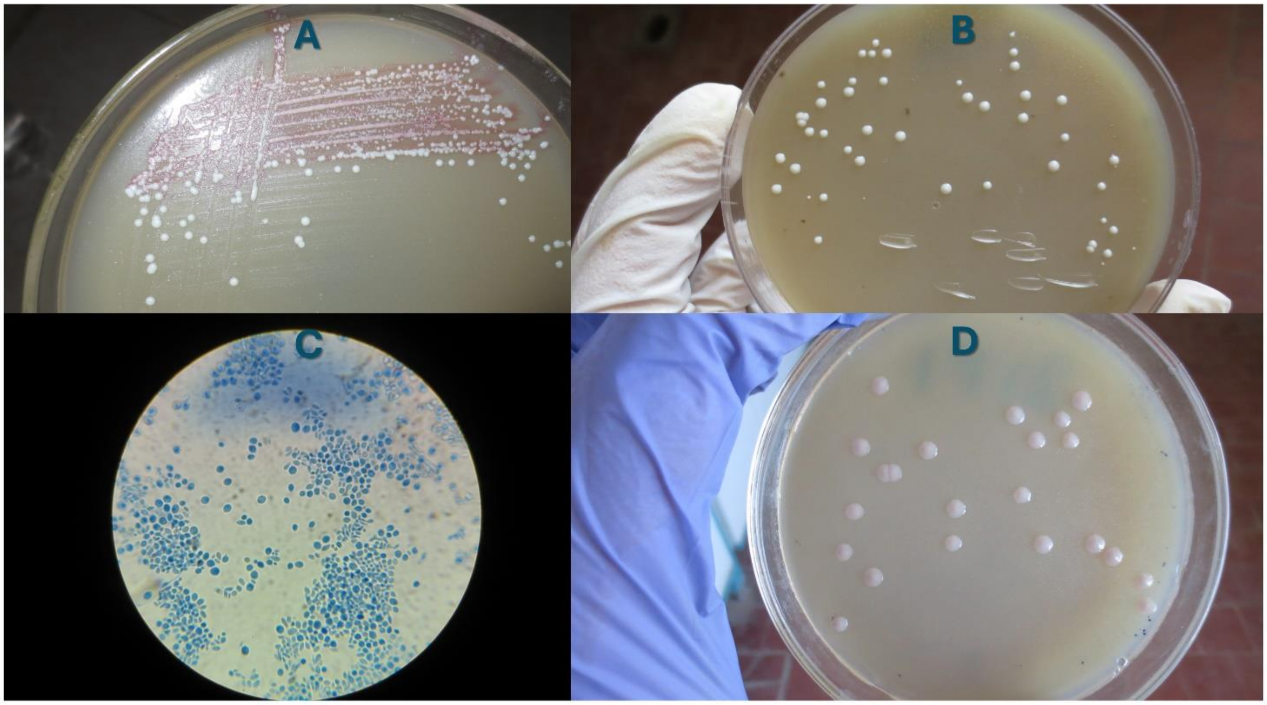

Candida Glabrata growth on CHROMagar Candida medium showing white smooth glossy soft colonies, sometimes pinkish (A-B-D). Microscopic morphology of *C. glabrata* at 100x magnification using lactophenol cotton blue showing Spherical to ovoid vegetative cells with small blastoconidia 1-4 um with no pseudohyphae (c)

Supplementary Figure 5: Macroscopic and Microscopic morphology of isolated *C. Krusei*

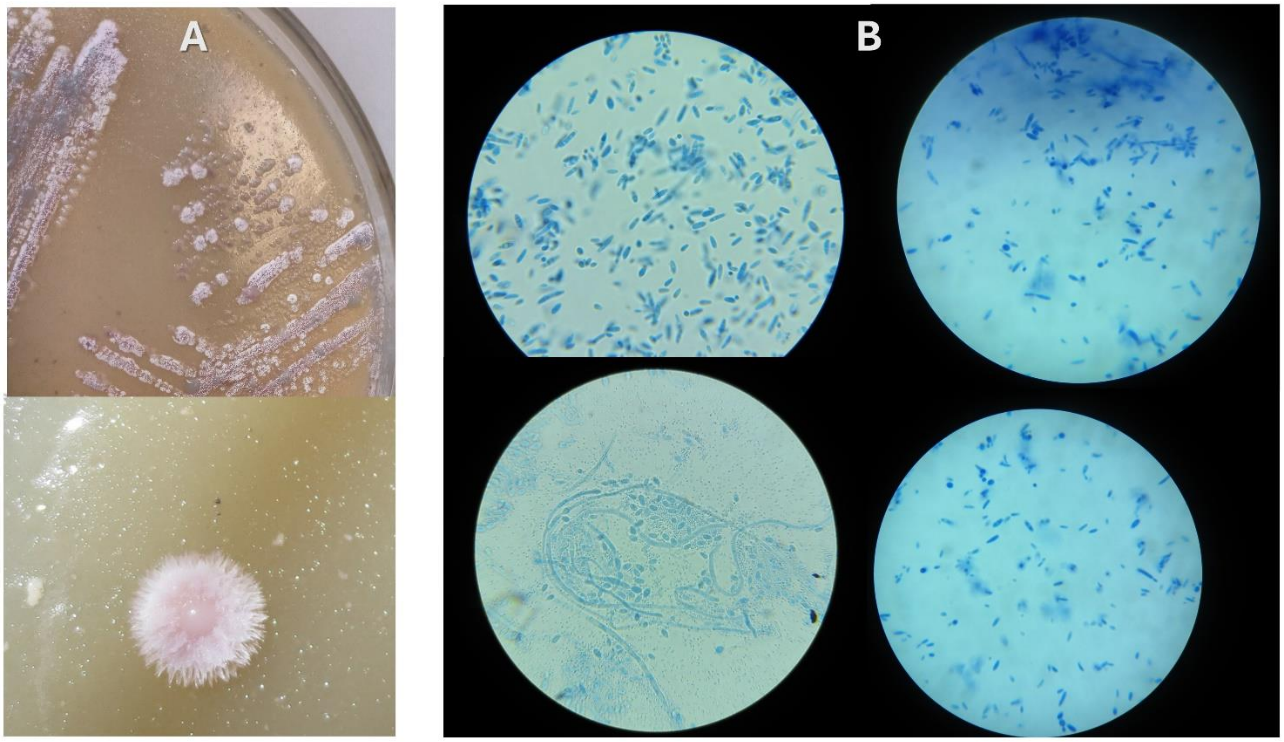

(A) Pale Pink rough fuzzy spreading broad colonies of *C. krusei* on CHROMagar Candida medium. (B) Microscopic morphology of *C. glabrata* at 100x magnification using lactophenol cotton blue showing small elongated to ovoid yeast cells with elongated blastoconidia resembling long grain rice and pseudohyphae.

Supplementary Figure 6: Macroscopic and Microscopic morphology of isolated *C. parapsilosis*

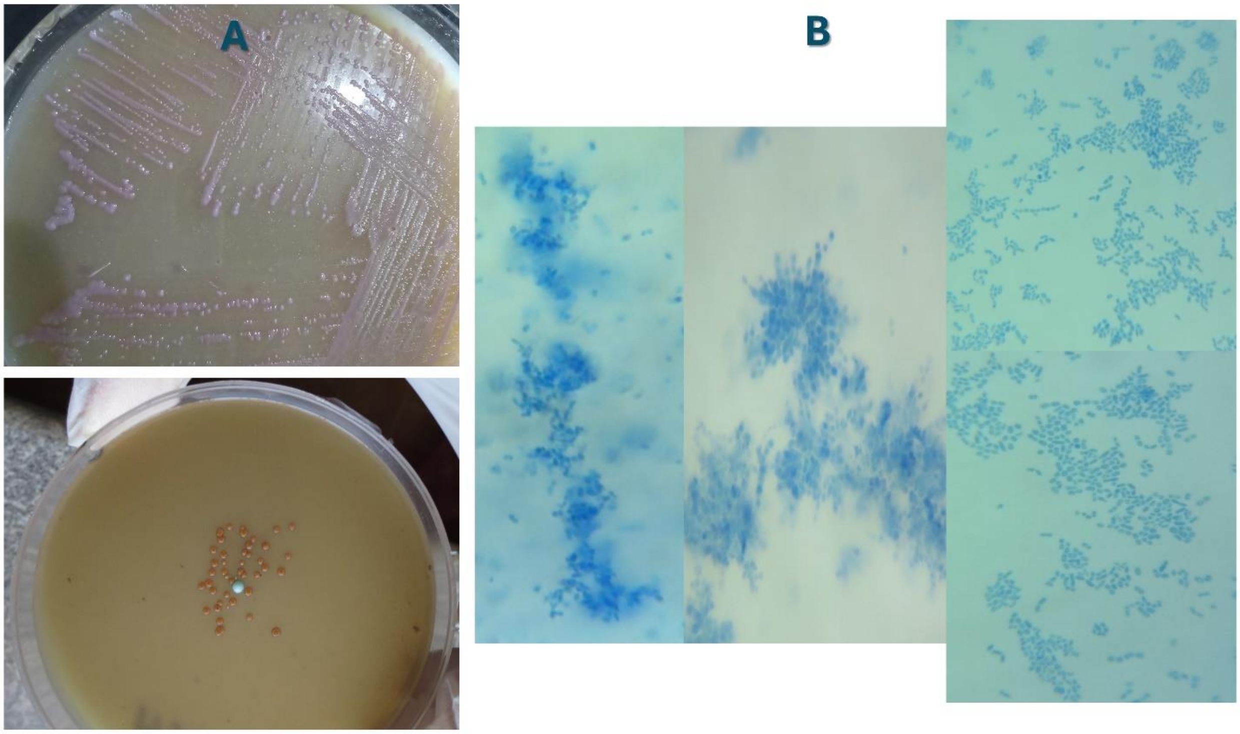

(A) Small pink to orange colonies of *C. parapsil*osis on CHROMagar Candida medium. (B) Microscopic morphology of *C. parapsilosis* at 100x magnification using lactophenol cotton blue showing small scattered blastoconidia (2.5–4μm) sometimes with spider radiating appearance

Supplementary Figure 7: Morphology of different isolated Candida species on Candida CHROMagar

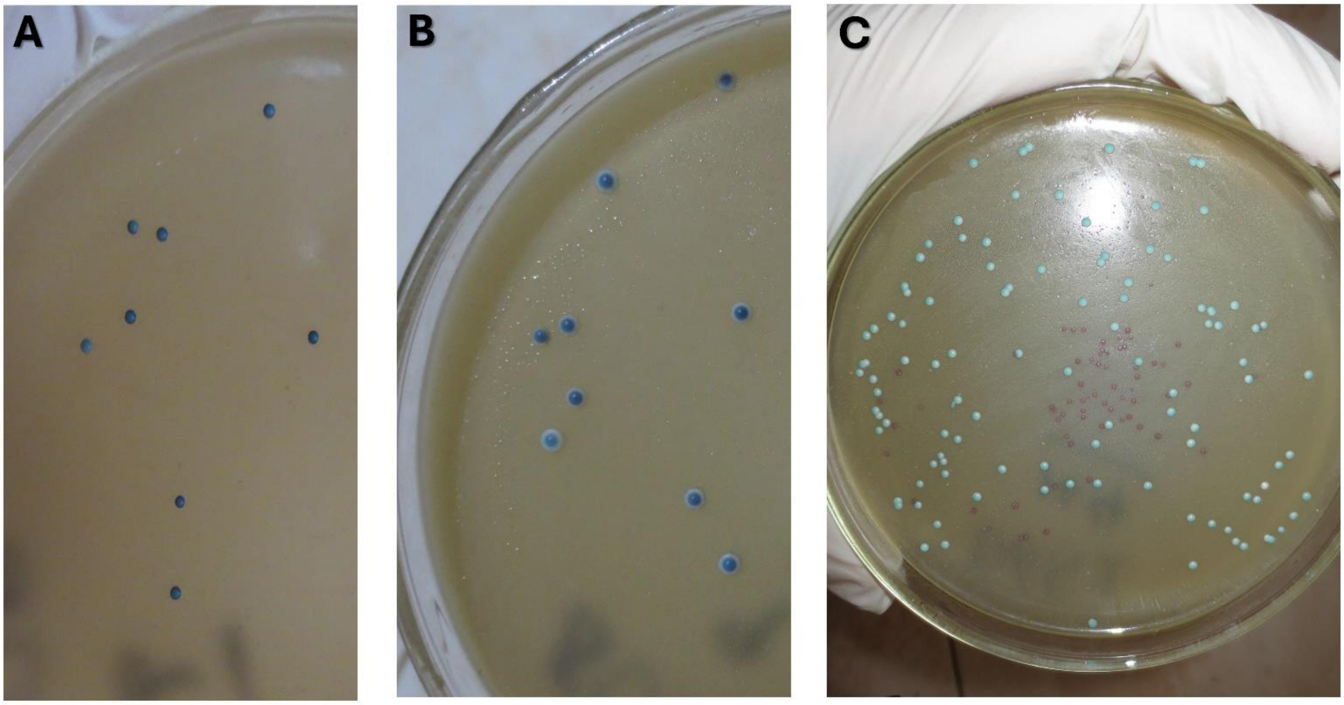

Candida tropicalis colonies on CHROMagar Candida medium showing violet to dark metallic blue colonies with paler edges (A-B).

Mixed Candida growth on CHROMagar Candida medium showing *C. albicans* and *C. parapsilosis* (C).

Supplementary Figure 8: Serial dilution cultures of *Lactobacilli* on Lactobacillus MRS agar

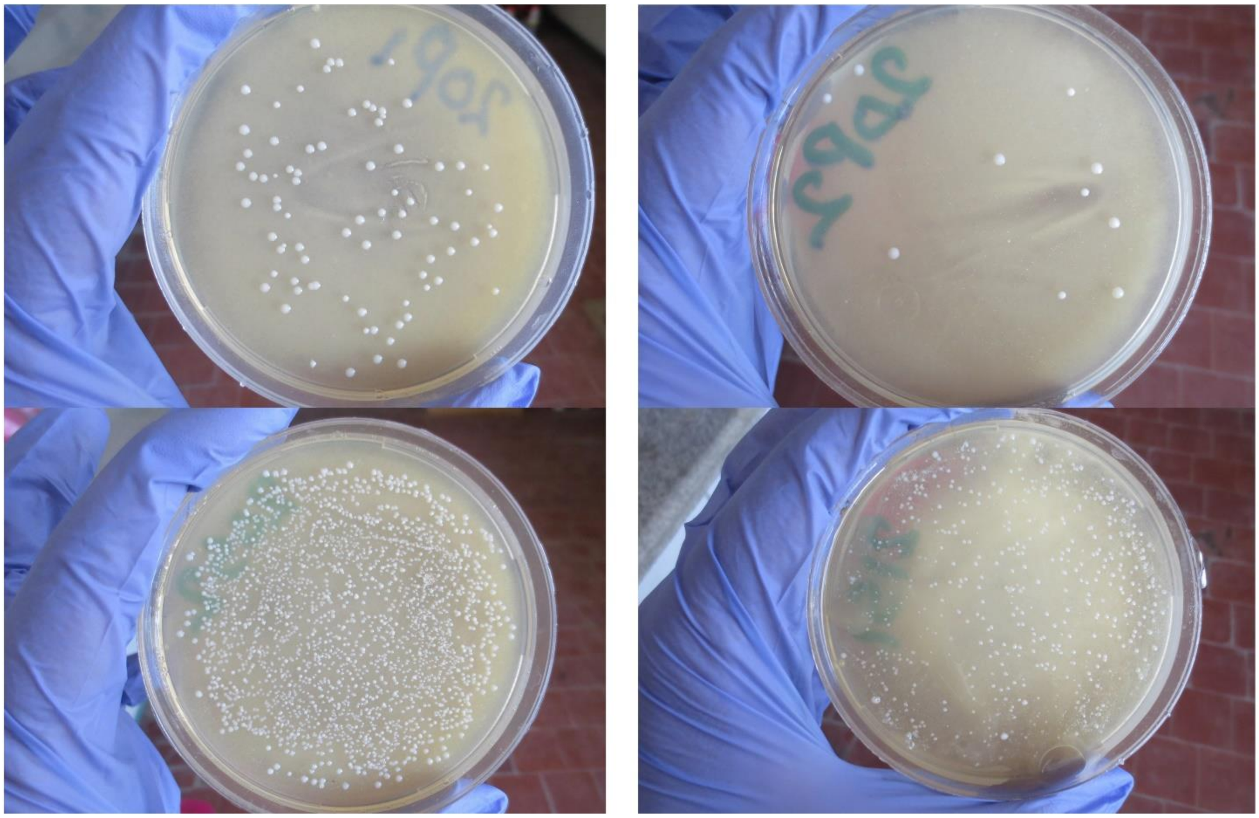

Supplementary Figure 9: Microscopic and Macroscopic morphology of isolated *Lactobacilli*

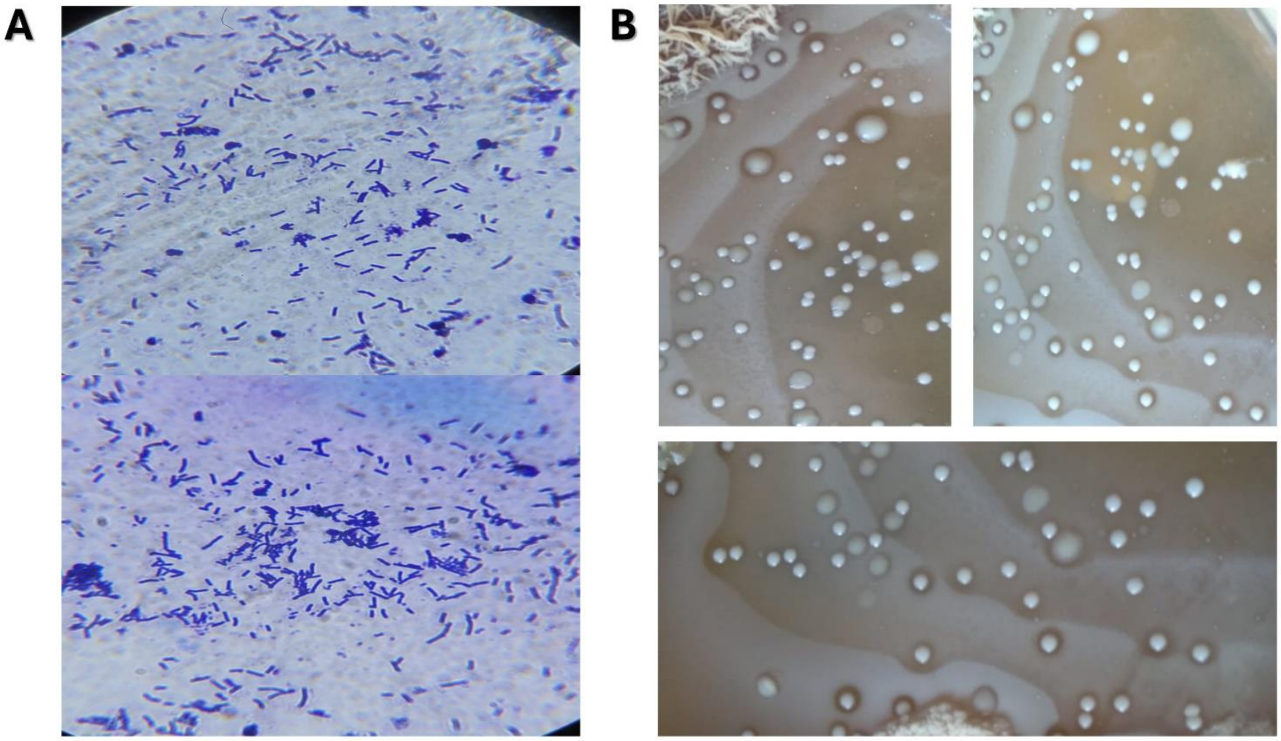

(A) Microscopic picture of *lactobacilli* showing Gram positive rods stained by crystal violet. (B) Colonies of *Lactobacilli* growing on MRS agar supplemented with 1.5% $CaCo_3$ showing clear zones of inhibition. *Lactobacillus* colonies were identified as milky white elevated moist circular colonies with clear zones

Supplementary Figure 10: Macroscopic and Microscopic morphology of Mutans Streptococci

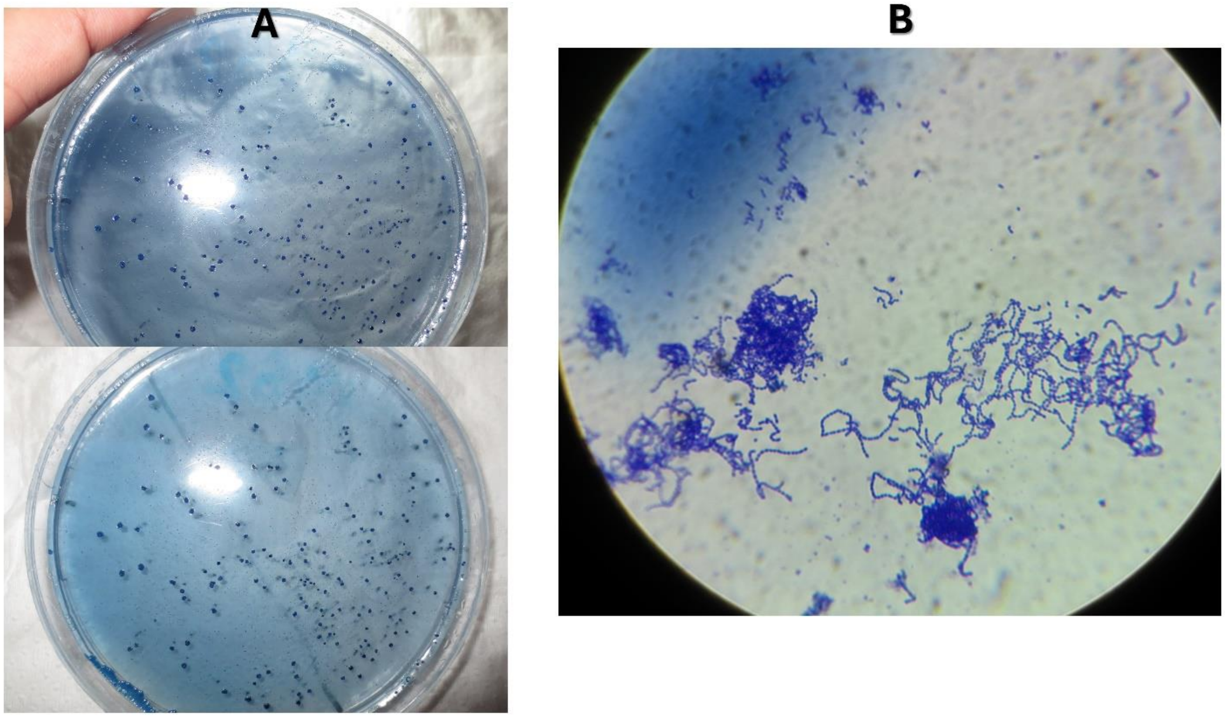

(A) Spread plate culture for enumeration of Mutans Streptococci on modified Mitis Salivarius agar. (B) Microscopic morphology of *Streptococcus mutans* showing Gram positive colonies arranged in chains.
